# CircRNAs Dysregulated in Juvenile Myelomonocytic Leukemia: CircMCTP1 Stands Out

**DOI:** 10.3389/fcell.2020.613540

**Published:** 2021-01-06

**Authors:** Anna Dal Molin, Mattias Hofmans, Enrico Gaffo, Alessia Buratin, Hélène Cavé, Christian Flotho, Valerie de Haas, Charlotte M. Niemeyer, Jan Stary, Pieter Van Vlierberghe, Jan Philippé, Barbara De Moerloose, Geertruij te Kronnie, Silvia Bresolin, Tim Lammens, Stefania Bortoluzzi

**Affiliations:** ^1^Department of Molecular Medicine, University of Padova, Padua, Italy; ^2^Department of Pediatric Hematology-Oncology and Stem Cell Transplantation, Ghent University Hospital, Ghent, Belgium; ^3^Department of Diagnostic Sciences, Ghent University Hospital, Ghent, Belgium; ^4^Department of Biology, University of Padova, Padua, Italy; ^5^Department of Genetics, University Hospital of Robert Debré, Paris, France; ^6^INSERM U1131, Institut de Recherche Saint-Louis, Université de Paris, Paris, France; ^7^Division of Pediatric Hematology and Oncology, Department of Pediatrics and Adolescent Medicine, University of Freiburg, Freiburg, Germany; ^8^Princess Máxima Center for Pediatric Oncology, Utrecht, Netherlands; ^9^Dutch Childhood Oncology Group, The Hague, Netherlands; ^10^Department of Pediatric Hematology/Oncology, Charles University and University Hospital Motol, Prague, Czechia; ^11^Cancer Research Institute Ghent, Ghent, Belgium; ^12^Center for Medical Genetics, Ghent University Hospital, Ghent, Belgium; ^13^Onco-Hematology, Stem Cell Transplant and Gene Therapy Laboratory, IRP-Istituto di Ricerca Pediatrica, Padua, Italy; ^14^Department of Maternal and Child Health, Padua University, Padua, Italy; ^15^Interdepartmental Research Center for Innovative Biotechnologies, University of Padova, Padua, Italy

**Keywords:** CircRNAs, regulatory networks, juvenile myelomonocytic leukemia, microRNAs, RNA-Seq

## Abstract

Juvenile myelomonocytic leukemia (JMML), a rare myelodysplastic/myeloproliferative neoplasm of early childhood, is characterized by clonal growth of RAS signaling addicted stem cells. JMML subtypes are defined by specific RAS pathway mutations and display distinct gene, microRNA (miRNA) and long non-coding RNA expression profiles. Here we zoom in on circular RNAs (circRNAs), molecules that, when abnormally expressed, may participate in malignant deviation of cellular processes. CirComPara software was used to annotate and quantify circRNAs in RNA-seq data of a “discovery cohort” comprising 19 JMML patients and 3 healthy donors (HD). In an independent set of 12 JMML patients and 6 HD, expression of 27 circRNAs was analyzed by qRT-PCR. CircRNA-miRNA-gene networks were reconstructed using circRNA function prediction and gene expression data. We identified 119 circRNAs dysregulated in JMML and 59 genes showing an imbalance of the circular and linear products. Our data indicated also circRNA expression differences among molecular subgroups of JMML. Validation of a set of deregulated circRNAs in an independent cohort of JMML patients confirmed the down-regulation of circOXNAD1 and circATM, and a marked up-regulation of circLYN, circAFF2, and circMCTP1. A new finding in JMML links up-regulated circMCTP1 with known tumor suppressor miRNAs. This and other predicted interactions with miRNAs connect dysregulated circRNAs to regulatory networks. In conclusion, this study provides insight into the circRNAome of JMML and paves the path to elucidate new molecular disease mechanisms putting forward circMCTP1 up-regulation as a robust example.

## Introduction

Juvenile myelomonocytic leukemia (JMML) is a rare and aggressive neoplasm of early childhood with a peak incidence around 2 years of age and an estimated incidence of approximately 1–2 per million (Niemeyer et al., 1997). Clonal growth of an abnormal multipotent hematopoietic stem cell leads to the characteristic proliferative features, such as monocytosis, splenomegaly and also a moderately elevated percentage of myeloblasts, while erythropoiesis and thrombopoiesis are mostly decreased (Niemeyer, 2014). Typical mutations in *NRAS* and *KRAS* (20–25%), *PTPN11* (35%), *NF1* (10–15%), or *CBL* (10–15%) genes can be found in 90–95% of patients and cause activation of the RAS signal transduction cascade. Within these genetically distinct subtypes, clinical and biological heterogeneity can be observed, from aggressive JMML needing hematopoietic stem cell transplantation in the majority of patients, to indolent disease with spontaneous resolution (Locatelli and Niemeyer, 2015). Clinical features such as age, hemoglobin F, platelet counts (Niemeyer et al., 1997) and gene expression signatures (Bresolin et al., 2010) were proven to have prognostic value. In recent years, development of the molecular biology toolbox has identified secondary mutations (Caye et al., 2015; Stieglitz et al., 2015; Coppe et al., 2018; Murakami et al., 2018), novel fusion genes (Lipka et al., 2017; Murakami et al., 2018; Chao et al., 2020), aberrant genomic DNA methylation (Lipka et al., 2017; Stieglitz et al., 2017; Murakami et al., 2018; Chao et al., 2020), and dysregulation in the non-coding transcriptome (microRNA, miRNA, and long non-coding RNA, lncRNAs, Leoncini et al., 2016; Hofmans et al., 2018) as molecular mechanisms associated with JMML pathogenesis and linked to prognostic relevance.

Circular RNAs (circRNAs) are single stranded RNA molecules with backspliced 3′- and 5′-ends linked in a non-collinear manner. Acting as miRNA sponges and competitive endogenous RNAs, circRNAs can indirectly regulate miRNA-target expression, ultimately controlling key miRNA-involving axes (Hansen et al., 2013; Li et al., 2015). CircRNAs can also interact with RNA-binding proteins (Schneider et al., 2016) and regulate cellular processes (Du et al., 2016). Several circRNAs were also shown to translate to unique peptides not encoded by linear transcripts (Legnini et al., 2017; Pamudurti et al., 2017; Yang et al., 2017). CircRNA-encoded peptides were shown to function in the regulation of cell differentiation (Legnini et al., 2017) and cancer progression (Rossi et al., 2019). More and more, the key role of circRNAs in leukemogenesis is emerging (Bonizzato et al., 2016; Jamal et al., 2019; Buratin et al., 2020) and perturbation of circRNA propagates in regulatory networks (Bhat et al., 2020) and signaling pathways, with pleiotropic effects.

Few studies have investigated circRNA expression in myeloid malignancies. In acute myeloid leukemia (AML), a role for circRNAs in the regulation of cell proliferation and leukemogenesis (Guarnerio et al., 2016; Sun et al., 2019; Yi et al., 2019), in drug resistance (Shang et al., 2019b; Cao et al., 2020), and as a diagnostic biomarker (Zhou et al., 2019) has been reported. Furthermore, mounting evidence suggests a role as potential therapeutic targets (Conn et al., 2015; Hirsch et al., 2017; Wu et al., 2018b; Bell et al., 2019; Shang et al., 2019a,b).

In this study, we provide the first data on the circRNA expression landscape in JMML patients identifying circMCTP1 ectopic expression as a new and possibly specific feature of JMML, further complementing the molecular base of JMML disease and paving the way to the identification of novel biomarkers and therapeutic targets.

## Materials and Methods

### Patients

Total RNA from diagnostic bone marrow (BM) or peripheral blood (PB) samples from 31 children with JMML was obtained from different institutions throughout Europe (Belgium, Netherlands, France, Czech, and Germany). None of the patients received prior treatment (Supplementary Table 1). Additionally, BM and PB from pediatric healthy donors (HD); (siblings screened for transplantation) were collected from 9 healthy subjects at the Ghent University Hospital (Supplementary Table 1). The “discovery cohort” used for RNA-seq profiling comprised 19 JMML and 3 HD. Results were validated in an additional cohort of 12 JMML patients and 6 HD. Out of 31 JMML patients, 15 are registered in the European Working Group of Myelodysplastic Syndromes in Childhood (EWOG-MDS) studies EWOG-MDS98 and EWOG-MDS2006 (National Institutes of Health trials registered as #NCT00047268 and #NCT00662090 at www.clinicaltrials.gov), and 16 in the French national JMML biobank. Written informed consent was obtained from the parents or legal guardians of patients before sample collection and in accordance with the Declaration of Helsinki. The study approval was granted from institutional review committees at each participating center.

### RNA Sequencing

Total RNA was extracted from isolated mononuclear cell preparations from BM or PB from 19 JMML patients (Supplementary Table 1), and 3 BM samples from HD. RNA was extracted using the miRNeasy Mini or Micro Kit (Qiagen) in combination with on-column DNase I digestion (RNase-Free DNase set, Qiagen) according to manufacturer’s instructions. RNA quality was assessed using the RNA 6000 Nano and RNA 6000 PICO assays on a 2100 Bioanalyzer (Agilent). Sequencing libraries were prepared using a TruSeq Stranded Total RNA Kit adapted for long fragments (±550 bp) with the Ribo-Zero Gold rRNA Removal Kit (Illumina) according to the manufacturer’s instructions. Prepared libraries were run on a HiSeq3000 high-throughput sequencing system (Illumina) and paired-end reads were generated (average of 52 million reads per sample).

### CircRNA Detection, Quantification and Filtering

Circular RNAs were identified and quantified from RNA-seq data by CirComPara v0.6.3 (Gaffo et al., 2017), using 9 backsplice detection methods (Supplementary Material).

CircRNAs reported by at least two methods with a raw count of at least five reads in at least one sample were considered detected. Only circRNAs expressed in at least one half of samples per condition (JMML and HD), or in over 34 of samples in at least one JMML subgroup were kept for the following analyses.

CircRNAs annotation and all analyses were based on the Ensembl GRCh38 human genome and annotation v93. Loci without annotated genes and expressing one or more circRNAs (overlapping or not far than 5,000 nt) defined new loci, called “CircClust.” Exon numbers (respectively, to the longest Ensembl transcript) are indicated in the circRNA names when multiple isoforms from the same gene are mentioned or to identify validated circRNAs.

Circular to linear proportion (CLP) was calculated as described in Supplementary Material.

### CircRNA Differential Expression

Circular RNAs expression was normalized with the regularized logarithm method (Love et al., 2014), and the surrogate variables were estimated with the *sva* R package (Leek, 2014) in order to remove hidden batch effects.

Differential expression in pairwise comparisons between conditions or sample groups was assessed by DESeq2 (Love et al., 2014) (v1.22.2) with local fit model including Wald significance tests, no independent filtering and correction for surrogate variables. *P*-values were corrected for multiple tests with the Benjamini Hochberg procedure, and an adjusted *p*-value ≤ 0.05 was chosen to detect significant differential expression.

### CircRNA-miRNA-Gene Network Reconstruction

Putative circRNA sequences were assembled joining annotated exons contained in the genomic coordinates corresponding to the validated backsplice ends. MiRNA binding sites were predicted through miRanda (John et al., 2004) (score ≥ 100 and free energy ≤ −20 kcal/mol) and PITA (Kertesz et al., 2007) (ΔG_*duplex*_ < −20 kcal/mol and ΔG_*open*_ > −13 kcal/mol), keeping only miRNAs with commonly predicted binding sites. MiRNAs were subsequently prioritized according to expression in a published series of 21 JMML patients [GEO Series accession number GSE71452 (Helsmoortel et al., 2016)]. For recently identified miRNAs, not quantified in the series, at least three predicted binding sites for the same circRNA were required.

Validated miRNA target genes (strong miRTarBase validation categories) were retrieved by Mienturnet web tool (Licursi et al., 2019). Gene expression of validated miRNA targets was obtained through StringTie v1.3.3 implemented in CirComPara. Genes with a mean TPM expression in JMML samples greater than 1 were considered expressed.

CircRNA-miRNA-gene networks were visualized using Cytoscape v3.7.2.

### Quantification of circRNAs by qRT-PCR

Synthesis of cDNA was performed after an additional in-solution gDNase elimination step (Heat&Run gDNA removal kit, ArcticZymes), using the 5x PrimeScriptTM RT Master Mix (Takara Bio Europe S.A.S.). All qRT-PCR reactions were carried out with custom designed primers (Supplementary Table 2) in 96-well plates using the Takyon qRT-PCR MasterMixes for SYBR assays (Eurogentec) on a Viia7 analyzer (ThermoFisher). *TBP*, *HPRT1* and *GAPDH* were used as internal reference genes. Ct thresholds were automatically determined by the QuantStudio^TM^ Real-Time PCR Software and data analysis was performed according to state-of-the-art methods. Briefly, Ct values generated for each target were corrected for primer pair efficiency and expressed as relative quantities (RQ). Normalized relative quantities (NRQ) were calculated by normalizing RQ values against the expression of housekeeping genes. Additional details are available in Supplementary Material.

## Results

### CircRNAs Expression in Patients With JMML and Healthy Donors

A cohort of 19 JMML patients representing the four main subtypes (8 *PTPN11*, 5 *KRAS*, 4 *NRAS*, and 2 *NF1* samples*;* Supplementary Table 1) was analyzed together with 3 age-matched healthy donors (HD; GEO ID: GSE147523) to study circRNA expression in patients with JMML.

**FIGURE 1:**
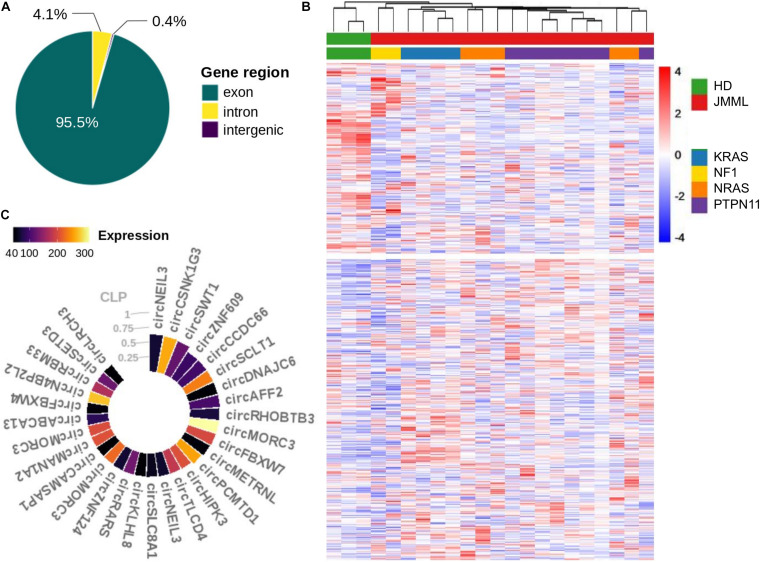
CircRNAs expressed in BM of JMML patients and healthy subjects. **(A)** Proportion of circRNAs derived from genic and intergenic regions; **(B)** Heatmap (row scaled values, clustering according to Euclidean distance) of normalized expression profiles of the 5,323 circRNAs considered in the study, corrected for surrogate variables; **(C)** CircRNAs with CLP ≥ 0.33 and circular mean expression > 40 in JMML.

Quantification and annotation of circRNAs, from high-depth ribo-depleted RNA-seq data, by CirComPara (Gaffo et al., 2017) detected 43,757 circRNAs, of which 5,323 with high expression were considered for further investigation (see section “Materials and Methods”). These circRNAs derived from 2,585 loci, mostly from exonic regions (95.5%), followed by gene introns and genomic regions without known genes (Figure 1A). Overall, 1,133 (43.8%) circRNA host genes expressed multiple circular isoforms each, with 28 genes expressing 10 up to 23 isoforms (Supplementary Figure 1 in Supplementary Material).

Clustering of circRNA expression profiles pointed toward differences between JMML patients and HD, separating circRNAs into two almost equally numerous groups of circRNAs with a tendency of over and under expression in JMML (Figure 1B).

Considering the circular to linear proportion (CLP, giving the relative expression of a circRNA in relation to the total of overlapping circular and linear transcripts) we identified 118 circRNAs accounting for at least one third of the total expression in JMML (CLP ≥ 0.33; Supplementary Table 3). Among the 30 with highest expression (Figure 1C), circCSNK1G3, circSCLT1 and circSWT1 were more abundant than their corresponding linear counterparts (CLP > 0.5), in JMML.

### CircRNAs Are Dysregulated in JMML

**FIGURE 2:**
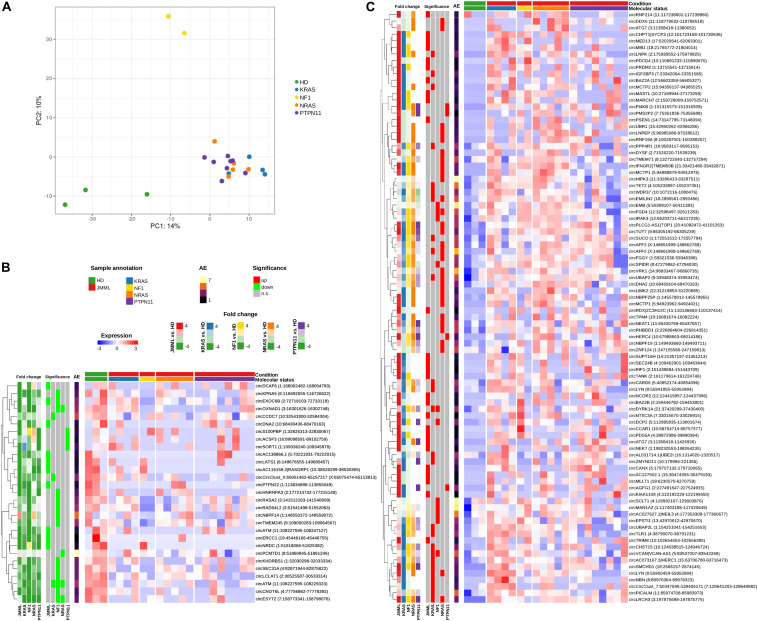
Significantly differentially expressed circRNAs in BM of JMML patients compared to HD. **(A)** Principal Component Analysis (PCA) of circRNA normalized expression profiles, corrected for surrogate variables; **(B,C)** Expression heatmaps of the 29 circRNAs down-regulated **(B)** and 90 up-regulated **(C)** in JMML. Expression given as row scaled values. The first 5 bars on the left of the heatmap show Log_2_(Fold Change) (“Fold change”), and the second 5 bars the significance of differential expression when comparing all JMML considered together and each JMML molecular subtype versus HD (“Significance”); “AE,” per row average of absolute expression.

Circular RNAs expression profiles separated JMML samples from HD according to unsupervised principal component analysis (PCA; Figure 2A), pointing toward a general dysregulation of circRNA expression in patients. Considerable heterogeneity was observed among JMML patients, and both PCA and clustering (Figure 1B) showed that *KRAS*, *NRAS*, and *PTPN11* mutated patients clustered together, while the two *NF1* samples clearly separated from both HD and the other molecular groups.

Comparing JMML with controls, we identified 119 circRNAs significantly differentially expressed (Figures 2B,C and Supplementary Table 4). Of those, 37 circRNAs had different expression levels in the entire JMML cohort compared to HD. Pairwise comparisons of individual JMML subgroups to HD revealed 29 circRNAs dysregulated in *KRAS*, 34 in *NRAS*, 7 in *PTPN11* and 25 in *NF1*, with 11 circRNAs dysregulated in two or three different JMML molecular subtypes. None of the circRNAs significantly dysregulated in *PTPN11* patients had significantly altered expression in one of the other three groups nor considering all JMML samples together. Nevertheless, it could be observed that several circRNAs significantly varied a single group showed a similar trend of expression in other groups, as exemplified by circKPNA5(3–12) which was significantly down-regulated in NF1 samples while simultaneously showing a generalized trend toward lower expression in the other molecular groups.

Of the 119 differentially expressed circRNAs, 90 were up-regulated in JMML compared to HD. Of these, 27 were identified considering the entire JMML cohort and 63 considering each subtype separately (Figure 2C). CircRNAs with a general marked up-regulation (log2FoldChange, LFC > 15) included circPSEN1, circCCAR1, and circMED13. In *KRAS* mutated JMML cases compared to HD, 26 circRNAs were up-regulated, including circPICALM, circTET2 and circZMYND11(2–4). In *NRAS*, 30 circRNAs were up-regulated, including circAFF2(2–3), circMCTP1(9–12), and circNBPF25P(13–14). Interestingly, circAGFG1, circHERC4, circPMS2P2, and circPI4KB were not expressed in control samples, while being up-regulated in the *PTPN11* subgroup. In *NF1*, 11 circRNAs were up-regulated, including circMAN1A2, circEMB and circSCLT1. Finally, we identified five circRNAs up-regulated in both *KRAS* and *NRAS* samples [circEMILIN2(4), circLRCH3, circLYN(5–8), circUBAP2, and circAFF2(3)], one in both *NF1* and *NRAS* (circFGD4), and two in both *KRAS* and *NF1* (circDYRK1A and circVCAN).

We detected 29 circRNAs down-regulated in JMML, of which 10 considering the whole cohort [particularly circATM(2–8), circPTPN22(8-intr14), and circLATS1(5–6)], and 19 considering subgroups of JMML separately (Figure 2B). CircEXOC6B turned out to be down-regulated in both comparisons. Three circRNAs were mutually down-regulated in different molecular groups: circATM(2–4) in *KRAS*, *NRAS* and *NF1* samples, circOXNAD1(7–8), and circESYT2(3–7) in both *NF1* and *NRAS*. CircX, derived from a gene in chromosome X likely expressing only circular transcripts (Gaffo et al., 2019), and circCCDC7 were down-regulated in *KRAS*. CircPCMTD1 was down-regulated in *NRAS*, while circS100PBP, circACSF3 and circSORT1 were less expressed in *PTPN11* samples than HD. The highest number of down-regulated circRNAs was observed in the *NF1* subgroup (*N* = 14) and included circEXOC6B.

Within the 119 differentially expressed circRNAs, we identified 6 genes with multiple circular isoforms. CircATM(2–8) was generally down-regulated in JMML, while circATM(2–4) was weakly expressed in all JMML subgroups except in *PTPN11* samples. CircATG7(13–18) was up-regulated in JMML altogether, circATG7(13–17) only in *NRAS* samples. CircMCTP1(7) and cirMCTP1(9–12) were both up-regulated in NRAS. Also, for *AFF2* and *LYN* genes two overlapping circular isoforms were identified: circAFF2(3) and circLYN(5–8) were up-regulated in both *KRAS* and *NRAS*, circAFF2(2–3) and circLYN(2–8) only in *NRAS* and *KRAS*, respectively. Surprisingly, an isoform switch was observed for the *DNA2* gene with circDNA2(2–14) generally more expressed in HD and circDNA2(2–5) isoform overexpressed in *NRAS* patients.

### Genes With Imbalances of Circular and Linear Transcripts in JMML

**FIGURE 3:**
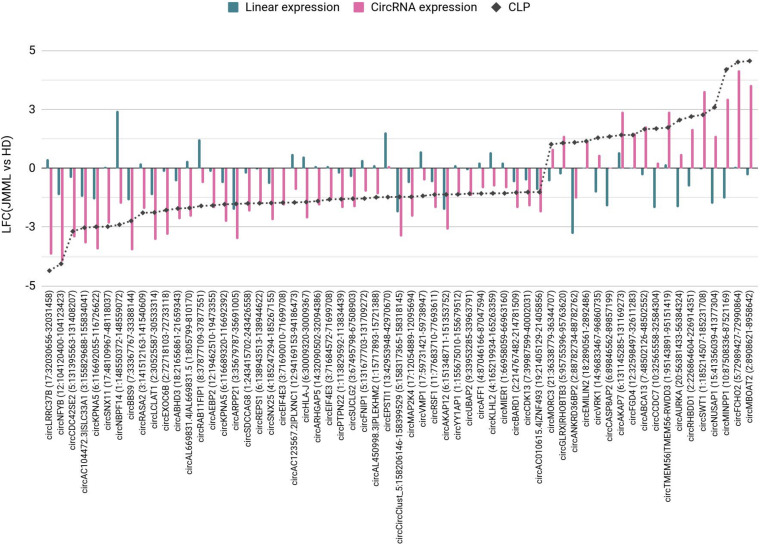
Circular to linear proportion (CLP) dysregulated in JMML compared to healthy donors. The 61 circRNAs with high absolute expression (normalized read count ≥ 100) and CLP (≥0.1) in JMML or HD for which the proportion of circular expression over the total (linear and circular) expression in JMML was at least two times increased or decreased compared to HD (|LFC(CLP)| ≥ 1). The plot shows the LFC observed comparing JMML with HD absolute linear and circular expressions (blue and pink bars, respectively) and CLP (dashed gray line). HD, healthy donors; LFC, log_2_(Fold Change).

Beyond absolute dysregulation of circRNA expression, also imbalances of circular and linear transcript expression in JMML emerged. Sixty-one circRNAs showed high absolute expression and varying CLP in JMML or in controls (Figure 3).

An increase of the CLP in JMML was observed for 18 circRNAs. This was due to a larger expression increase of the circRNA than the linear counterpart [e.g., circFCHO2, circEMILIN2(4), circAKAP7, circSWT1, and circTMEM56], or to a decrease of the linear transcripts (e.g., circNUSAP1 and circMINPP1). The decrease of circANKRD36B with a more marked decrease of the linear counterpart resulted in a relative increase of this circRNA in JMML. For 53 circRNAs the CLP decreased comparing JMML with HD, mostly due to a concordant behavior of circular and linear transcripts, with a more conspicuous decrease of the circRNA (e.g., circNFYB). CircEXOC6B was significantly down-regulated in JMML, whereas the linear transcripts of its host gene were unvaried, thus circEXOC6B CLP was higher in controls (0.62) than in JMML (0.18). Additionally, the CLP decrease for circNBPF14 in JMML reflected both a decrease of the circRNA and a marked increase of the linear counterpart. Three circRNAs had a very stable expression comparing JMML and controls, but a varied CLP due to the increase (circEPSTI1) or to the decrease (circCASP8AP2 and circCCDC7) of their linear counterpart.

Of note, 9 circRNAs significantly differentially expressed in JMML (Figures 2B,C); the [up-regulated circEMILIN2(4), circVRK1, circFGD4, and circRHBDD1, and the down-regulated circNBPF14, circRASA2, circLCLAT1, circEXOC6B, and circKPNA5(3–12)] compared with HD were among the 61 with more pronounced variation of CLP (Figure 3). The altered expression of these circRNA did not co-occur with a corresponding variation of the linear counterpart and reflects an imbalance of the circular and linear products of the same gene.

### CircRNA Dysregulation Confirmed in a Validation Cohort

**FIGURE 4:**
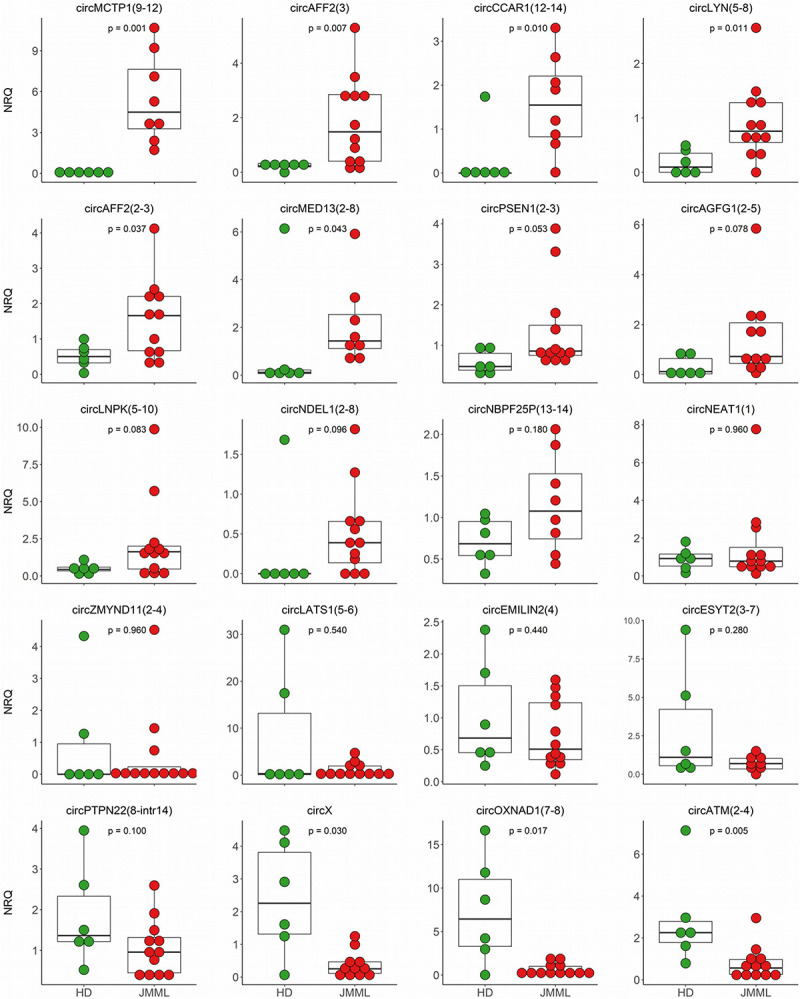
CircRNA differential expression validations. QRT-PCR quantifications of 20 circRNAs, with differential expression between JMML and HD according to RNA-seq data, were obtained in the validation cohort comprising 12 JMML (red dots) and 6 age-matched HD samples (green dots). CircRNA quantification in not less than 8 patients was obtained for all the circRNAs; *p*-values (two-tailed) were calculated by the Mann–Whitney *U* test.

Next, we selected 27 circRNAs with dysregulated expression in JMML patients for quantification in an independent validation cohort of 12 JMML samples and 6 HD (Supplementary Table 2). For all of the tested circRNAs the backsplicing sequence could be confirmed by Sanger sequencing. For 20 circRNAs, qRT-PCR quantification in the validation cohort strengthened our findings (Supplementary Table 1 and Figure 4).

Of note, for 9 circRNAs, the significant dysregulation in JMML patients observed by RNA-seq was confirmed in the validation cohort. CircATM(2–4), circOXNAD1(7–8), and circX were consistently down-regulated in JMML. CircCCAR1(12–14), circMED13(2–8), circMCTP1(9–12), circLYN(5–8), circAFF2(3), and circAFF2(2–3) were up-regulated. Most striking was the >80 times up-regulation of circMCTP1(9–12) in JMML. For five additional circRNAs, the same trend was observed in the two cohorts, with expression differences resulting marginally significant (0.05 < p.adj ≤ 0.1) in the validation cohort: circLNPK(5–10), circNDEL1(2–8), circPSEN1(2–3), and circAGFG1(2–5) showed a tendency toward up-regulation in JMML, circPTPN22(8-intr14) toward a general down-regulation. For the remaining 6 circRNAs, data of PCR-based quantification showed the same trend of expression observed by RNA-seq, without reaching the significance.

Expansion of the independent validation cohort with 9 patients from the discovery cohort with sufficient RNA available for qRT-PCR, depicted comparable and stable results, confirming the dysregulated expression of 15 circRNAs (Supplementary Figure 2 in Supplementary Material).

As circRNAs are well-known for decoying miRNAs, we evaluated the circRNA-miRNA-gene network for the five most markedly dysregulated circRNAs [circMCTP1(9–12), circAFF2(3), and circLYN(5–8) up-regulated; circATM(2–4) and circOXNAD1(7–8) down-regulated] (Supplementary Figure 3 in Supplementary Material).

### CircRNA Expression Variation in JMML Molecular Subtypes

Although the cohort of samples in each subgroup was limited, circRNA with expression differences among molecular subgroups of JMML emerged from our data.

**FIGURE 5:**
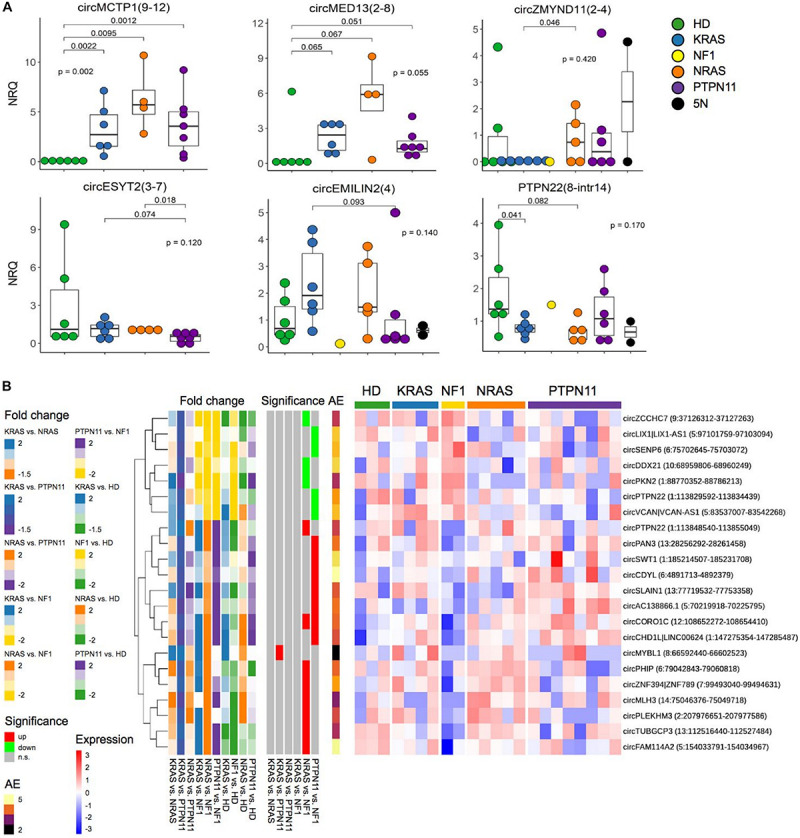
CircRNA expression variation in molecular subtypes of JMML. **(A)** QRT-PCR expression data of 6 circRNAs in 21 JMML of 5 molecular subtypes [*KRAS*, *NRAS*, *PTPN11* and *NF1* mutated, and quintuple negative (5N)], and 6 age-matched HD samples. CircRNA quantification in not less than 17 patients was obtained for all the circRNAs; differential expression of *KRAS*, *NRAS*, and *PTPN11* groups compared with HD (Kruskal–Wallis test) and to each other was tested with Mann–Whitney *U* test; only *p*-values < 0.1 are shown for the corresponding pairwise comparison. **(B)** Expression heatmap of circRNAs differentially expressed between JMML molecular subtypes according to RNA-seq data (sample expression is given as row scaled values). The first group of bars on the left of the heatmap show Log_2_(Fold Change) comparing *KRAS*, *NRAS*, and *PTPN11* groups to each other and with HD (“Fold change”); the second group of bars show the significance of differential expression in pairwise JMML subgroups comparisons (“Significance”); “AE,” per row average of absolute expression.

QRT-PCR validations of the panel of 20 circRNAs scrutinized for differential expression in JMML suggested significant, even if not sizable, variation of circRNA expression among groups (Figure 5A and Supplementary Figure 4 in Supplementary Material). CircMCTP1(9–12) and circMED13(2–8) up-regulation was more marked in *NRAS*; circZMYND11(2–4) was more expressed in *NRAS* than in *KRAS* samples; circESYT2(3–7) showed down-regulation in *PTPN11* compared to *NRAS*, and at some extent also to *KRAS*; circEMILIN2(4) had a tendency of up-regulation in *KRAS* compared to *PTPN11* and circPTPN22(8-intr14) was down-regulated in both *KRAS* and *NRAS*, but not in *PTPN11*, compared to controls.

Following these observations, RNA-seq based direct pairwise comparisons between groups identified 22 additional circRNAs significantly varying between groups (Figure 5B). Apart from circMYBL1, which showed significantly lower expression in *PTPN11* compared with *KRAS*, all the other circRNAs (7 up-regulated and 14 down-regulated) significantly varied between *NF1* patients and the other groups. Noteworthy, circVCAN was significantly more expressed in *NF1* compared with *PTPN11*, and significantly up-regulated comparing *NF1* and *KRAS* JMML subtypes with HD (Figure 2).

### CircMCTP1 Dysregulation in JMML

CircMCTP1(9–12) was found to be highly up-regulated in JMML compared with HD (Figure 4 and Supplementary Figure 2) and with higher levels observed in NRAS patients, consistently according to RNA-seq (Figure 2C) and qRT-PCR data in the extended cohort (Figure 5A).

CircMCPT1 dysregulation is a completely new finding and the function of this circRNA is still unknown. Considering that one of the prominent functions assigned to circRNAs is to decoy miRNAs thus interfering with they regulatory activity, we further investigated the possible interactions of circMCTP1 with miRNAs, and reconstructed circRNA-miRNA-gene networks that informed the possible function of circMCTP1 in JMML.

**FIGURE 6:**
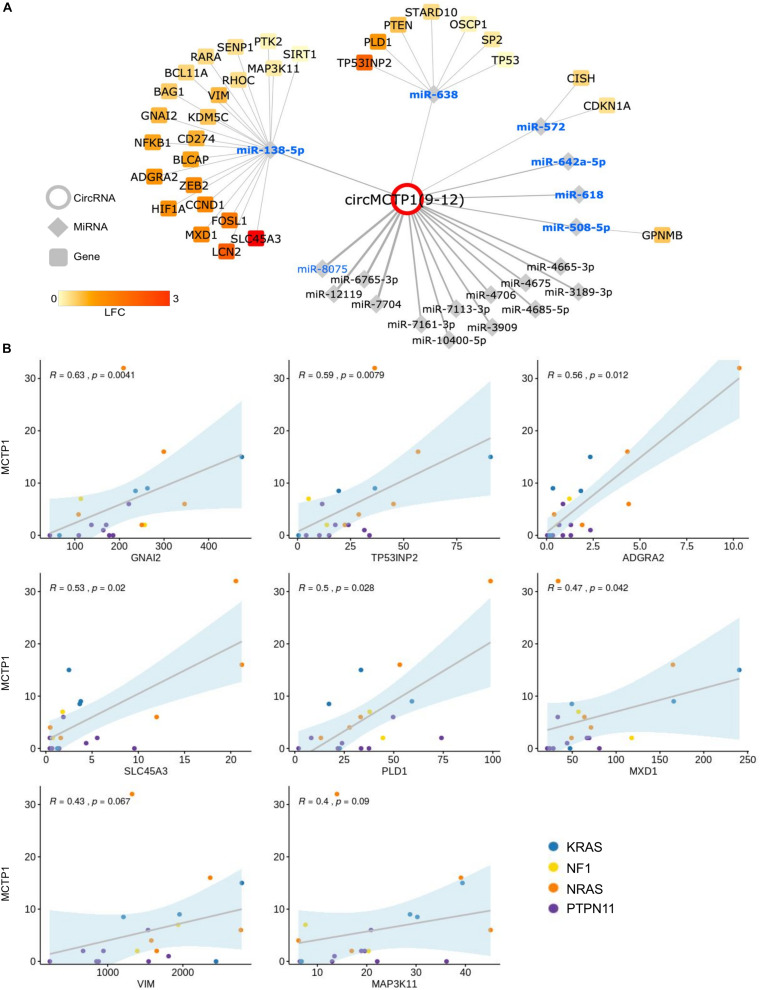
Predicted circMCTP1-miRNA-gene network. **(A)** CircMCTP1 (red circle) is linked to miRNAs (diamonds) according to binding sites predicted by both miRanda and PITA tools; names in bold and blue indicate, respectively, miRNAs expressed in JMML (Leoncini et al., 2016) and with tumor suppressor role according to literature data; recently identified miRNAs with at least three predicted binding sites are also reported; size of circMCTP1-miRNA edges is proportional to the number of the predicted binding sites for that miRNA (range 1–4); validated miRNA target genes resulting from Mienturnet (strong MiRTarBase categories) are shown as squares, with fill color indicating the LFC of the gene expression variation comparing JMML with HD. **(B)** In JMML patients expression of eight genes in the network was strongly correlated (ρ ≥ 0.4) with circMCTP1 level.

Interestingly, circMCTP1 harbors predicted binding sites for well-known tumor suppressor miRNAs (Figure 6A), including miR-8075 (Song et al., 2019) for which four binding sites were found in the circRNA sequence, and several miRNAs known to be expressed in JMML according to Leoncini et al. (2016). Further, consistent with the hypothesis that circMCTP1 aberrant up-regulation could de-repress specific miRNA-target genes in malignant cells, our analysis of RNA-seq gene expression data in the discovery cohort identified increased expression in JMML of validated targets of circMCTP1-associated miRNAs. Particularly, 32 target genes of miR-138-5p, miR-638 (Lin et al., 2015), miR-508, and miR-572 had increased expression in JMML, including eight that also showed, in JMML patients, an expression profile strongly positively correlated (ρ ≥ 0.4) with circMCTP1 expression (Figure 6B). These data pointed at possible regulatory axes impacted by circMCTP1 up-regulation.

## Discussion

Knowledge of circRNA involvement in pathogenetic mechanisms of cancer (Kregel et al., 2019) and leukemia, such as AML (Guarnerio et al., 2016; Sun et al., 2019; Yi et al., 2019) and acute lymphoblastic leukemia (ALL) (Hirsch et al., 2017; Gaffo et al., 2019; Molin et al., 2019) (make circRNAs extremely attractive molecules for translational research in oncohematology (Bonizzato et al., 2016; Kristensen et al., 2018)). In this study we used total RNA sequencing to uncover the circRNA transcriptome in 19 JMML patients of four molecular subtypes and their potential role in JMML disease.

We showed that JMML samples like all other human tissues studied so far possess a wide array of circRNAs and more importantly, that the circRNAome is altered comparing JMML patients to HD. In total, 119 circRNAs were differentially expressed of which 37 significantly taking all JMML samples together and 83 when comparing each molecular subtype with HD (29 dysregulated in *KRAS*, 34 in *NRAS*, 7 in *PTPN11* and 25 in *NF1*).

A few circRNAs dysregulated in JMML were reported earlier as aberrantly expressed in other types of cancer. Down-regulation of circEXOC6B in JMML, particularly in *NF1* patients, had been detected in epithelial ovarian cancer in association with a lower survival rate (Ning et al., 2018). Up-regulation in JMML of the oncogenic circHIPK3 (Zheng et al., 2016; Chen et al., 2020) is in line with reported overexpression in B-cell precursor ALL (Gaffo et al., 2019) and in chronic myeloid leukemia (Feng et al., 2020). Two other circRNA up-regulated in JMML were linked to breast cancer, circIRAK3 (Wu et al., 2018a) and circEPSTI1 (Chen et al., 2018). The latter and circMAN1A2 (Fan et al., 2019) were previously proposed as biomarkers of malignancy. CircDYRK1A down-regulation, observed in JMML, was found in plasma of patients with gastric cancer (Chen et al., 2017). Additional studies are needed to prove, in JMML, the oncogenic role of these and other still less characterized circRNAs that resulted dysregulated in our data.

Robust evidence about dysregulation of 15 circRNAs was obtained by analysis of two independent cohorts. CircOXNAD1(7–8) and circATM(2–4) were consistently down-regulated in JMML patients compared to HD in the two cohorts. CircOXNAD1(7–8) was previously shown to be more expressed in monocytes compared with lymphocytes (Gaffo et al., 2019). CircATM(2–4), derived from the *ATM* gene, encoding a protein important for DNA repair whose mutations are found in myeloid malignancies (Ganguly and Kadam, 2016), was the most abundant circRNA in BM of healthy donors and showed a generalized down-regulation in JMML.

CircMCTP1(9–12), circLYN(5–8), and circAFF2(3) were the most highly up-regulated circRNAs in JMML. None of these had been heretofore identified in a myeloid malignancy. CircAFF2(3) is overexpressed also in B-cell precursor ALL (Gaffo et al., 2019). In the same study, we have shown that, in normal haematopoiesis, both circMCTP1(9–12) and circAFF2(3) are up-regulated in monocytes compared to lymphocytes, whereas circLYN(5–8) is highly expressed both in monocytes and B-cells. Of note, up-regulation in JMML of circMCTP1(9–12), circLYN(5–8), and circAFF2 isoforms was not associated with a comparable increase of the linear counterpart: their CLP increase in JMML indicated that the circRNA variation was not a mere consequence of up-regulation of the corresponding host genes. This study identified, nevertheless, specific genes with an imbalance of circular and linear transcripts in JMML, detecting circRNAs with CLP variation in JMML compared with controls, such as the increase of circFCHO2 and circSWT1 and the decrease of circNBPF14 CLP. Of note, we discovered that nine of the circRNAs significantly differentially expressed in JMML presented also an imbalance of the circular and linear products of the gene. Regulation of circular RNA expression independent of linear expression is mainly uncharted territory. Our observations support the view that abnormal circRNA expression is often independent of linear expression variation.

Our finding of strong circMCTP1(9–12) up-regulation in JMML is completely new and constitutes an important highlight of this study. To our knowledge, circMCTP1(9–12) was not previously characterized nor reported. The host gene *MCTP1* (mast cell protease 1) has not been associated with hematological or solid malignancies. CircMCTP1(9–12) expression is potentially important for JMML pathogenesis and calls for further research. Functional predictions and gene expression data provided a first look at the complex circRNA-miRNA-gene networks that might contribute to JMML disease. CircRNA up-regulation was previously shown to restrain miRNA activity causing de-repression of miRNA target genes (Memczak et al., 2013; Shang et al., 2019b). In particular, predictions point at circMCTP1 potential to decoy tumor suppressor miRNAs expressed in JMML (Leoncini et al., 2016): miR-138-5p (Manafi Shabestari et al., 2018; Rastgoo et al., 2018), miR-638 (Lin et al., 2015), miR-508-5p (Chan et al., 2016), miR-572 (Guan et al., 2018), and miR-8075 (Song et al., 2019) for which four binding sites were predicted and for which sponging by circular or linear transcripts has been recently linked to cancer development (Song et al., 2019). Furthermore, amongst the validated target genes of circMCTP1 associated miRNAs, we identified genes with increased expression in JMML. These include the ZEB2 transcription factor essential for maintenance of leukemic growth in AML (Li et al., 2017), *LCN2* lipocalin whose ectopic expression promotes leukemogenesis (Chakraborty et al., 2012), and other genes previously linked to AML (*RARA*, *CCND1*, and *NFKB1*). In addition, up-regulated genes of the RAS signaling pathway (*IGFR1*, *NFKB1*, and *PLD1*), of the PD-L1 expression and PD-1 checkpoint pathway (*HIF1A*, *LCN2*, and *NFKB1*), and of the HIF-1 signaling pathway (*HIF1A* and *NFKB1*) were conspicuous in the circMCTP1 network. Among genes further highlighted by the observation of an expression profile, in JMML patients, positively correlated with circMCTP1 expression, *GNAI2, PLD1, ADGRA2, MXD1, VIM*, and *MAP3K11* are particularly noteworthy. In conclusion, analysis of networks linked ectopic circMCTP1 expression in JMML to miRNAs and to up-regulated target genes with oncogenic associations, suggesting new regulatory axes.

Our aim was to provide an overview of circRNA dysregulation in JMML. This knowledge is entirely new to the field and represents the starting point for further studies. We suggest that experimental studies are needed to clarify the functional roles of circRNAs in JMML and to shed light on involved mechanisms. Evidence collected by our analyses informed circRNA expression variation between JMML molecular subtypes and contribution to disease heterogeneity. On the level of circRNA expression, the *NF1* subgroup is more divergent from the other subgroups, in line with data about lncRNAs (Hofmans et al., 2018). This observation, and more subtle differences between *PTPN11* compared with *KRAS* and *NRAS*, calls for further investigation in larger cohorts, with also the potential to inform association of circRNA expression with biological and clinical features of patients, useful to establish new biomarkers in JMML. Finally, we propose that our data can pave the way to novel therapeutic strategies, possibly targeted to circRNA-involving mechanisms, for instance by implementing *in vivo* circRNA silencing strategies (Holdt et al., 2018).

In conclusion, we performed a comprehensive circRNA profiling in JMML, and demonstrated that JMML patients harbor a distinct circRNA expression profile compared to healthy controls. Dysregulation of known and many unknown circRNAs, such as circMCTP1, suggests a role for circRNAs in JMML pathogenesis, and results of functional predictions give insights in the genes and biological pathways involved. This study offers a new view in JMML and paves the way for functional research on the impact of circRNAs dysregulation in JMML biology and their diagnostic and therapeutic application.

## Data Availability Statement

The datasets presented in this study can be found in online repositories. The names of the repository/repositories and accession number(s) can be found below: https://www.ncbi.nlm.nih.gov/geo/, GSE147523 and GSE71452.

## Ethics Statement

The studies involving human participants were reviewed and approved by Out of 31 JMML patients, 15 are registered in the European Working Group of Myelodysplastic Syndromes in Childhood (EWOG-MDS) studies EWOG-MDS98 and EWOG-MDS2006 (National Institutes of Health trials registered as #NCT00047268 and #NCT00662090 at www.clinicaltrials.gov), and 16 in the French national JMML biobank. Written informed consent was obtained from the parents or legal guardians of patients before sample collection and in accordance with the Declaration of Helsinki. The study approval was granted from the institutional review committee at the Ghent university hospital (EC/2011/825) and at each participating center. Written informed consent to participate in this study was provided by the participants’ legal guardian/next of kin.

## Author Contributions

AD, MH, PV, GK, SBr, TL, and SBo conceived the study. AD, EG, AB, and SBo contributed bioinformatics methods and data analysis. MH and TL provided RNA-seq data and performed experimental validations. AD, MH, SBr, TL, and SBo drafted the manuscript. AD, MH, and SBo made the figures. HC, CF, VH, CN, JS, JP, and BD contributed patient samples. All authors revised the manuscript.

## Conflict of Interest

The authors declare that the research was conducted in the absence of any commercial or financial relationships that could be construed as a potential conflict of interest.

## Abbreviations

JMML: Juvenile Myelomonocytic Leukemia
miRNA: microRNA
lncRNA: long non-coding RNA
circRNA: circular RNA
RNA-seq: RNA sequencing
HD: healthy donors
qRT-PCR: Quantitative Reverse Transcription Polymerase Chain Reaction
HSCT: hematopoietic stem cell transplantation
AML: Acute Myeloid Leukemia
BM: bone marrow
PB: peripheral blood
CLP: Circular to Linear Proportion
TPM: transcripts per million
PCA: Principal Component Analysis
LFC: log_2_(Fold Change)
ALL: Acute Lymphoblastic Leukemia.
