# Supplementary material for: CircRNAs Dysregulated in Juvenile Myelomonocytic Leukemia: CircMCTP1 Stands Out
Source: Front Cell Dev Biol. 2021 Jan 6;8:613540. doi: 10.3389/fcell.2020.613540 (PMC7815690; doi:10.3389/fcell.2020.613540)

**Supplementary Methods and Figures**

[**Supplementary M**](#_xacdlocailp)**ethods 2**

[CircRNA detection and quantification](#_c3m389icx9n6) 2

CircRNA quantification by qRT-PCR

[**Supplementary F**](#_n2zg6njszz8i)**igures 6**

[Supplementary Figure 1. Genes with at least 10 circular isoforms.](#_i3wr64rnna3g) 6

[Supplementary Figure 2. Expression variation in 21 JMML cases of the circRNA panel quantified by qRT-PCR.](#_tnwmx9srpjdf) 8

[Supplementary Figure 3. Predicted circRNA-miRNA-gene network involving circRNAs most deregulated in JMML.](#_skqfzmpk9hg0) 8

[Supplementary Figure 4. Expression variation in 21 JMML of different molecular subtypes of the circRNA panel quantified by qRT-PCR.](#_v3zafn874jsu) 10

#

#

# Supplementary Methods

## CircRNA detection and quantification from RNA-seq

CircRNAs were detected and quantified by CirComPara v0.6.3[(Gaffo et al. 2017)](https://paperpile.com/c/0nC3Wy/m7369) using 9 backsplice detection methods (CIRI2 v2.0.2[(Gao, Zhang, and Zhao 2018)](https://paperpile.com/c/0nC3Wy/BWfYd); Findcirc v1.2[(Memczak et al. 2013a, [b] 2013)](https://paperpile.com/c/0nC3Wy/9dAvR+aVswp); circRNA_finder v.1.1; DCC v0.4.6 [(Cheng, Metge, and Dieterich 2016)](https://paperpile.com/c/0nC3Wy/ojclF); CIRCexplorer2 v2.3.3[(Zhang et al. 2016)](https://paperpile.com/c/0nC3Wy/KweOS) combined to each of BWA[(H. Du and Liang, n.d.)](https://paperpile.com/c/0nC3Wy/2Jquo), STAR[(Dobin et al. 2013)](https://paperpile.com/c/0nC3Wy/1akAa), Segemehl[(Gao, Zhang, and Zhao 2018)](https://paperpile.com/c/0nC3Wy/BWfYd) and TopHat2[(Kim et al. 2013)](https://paperpile.com/c/0nC3Wy/SVY8j) alignments) and default parameters, which selected only the circRNAs detected by at least two methods.

Version of other software tools included in CirComPara: Bowtie2 v2.2.9[(Langmead and Salzberg 2012)](https://paperpile.com/c/0nC3Wy/ldpQq), BWA v0.7.15-r1140[(H. Li 2013)](https://paperpile.com/c/0nC3Wy/qoTe8), STAR v2.6.1d[(Dobin et al. 2013)](https://paperpile.com/c/0nC3Wy/1akAa), Segemehl v0.3.4[(Hoffmann et al. 2014)](https://paperpile.com/c/0nC3Wy/yhQq9), and TopHat2 v2.1.0[(Kim et al. 2013)](https://paperpile.com/c/0nC3Wy/SVY8j) with Bowtie v1.1.2[(Langmead et al. 2009)](https://paperpile.com/c/0nC3Wy/GLGFV).

CirComPara preprocessed raw reads with Trimmomatic v0.38[(Bolger, Lohse, and Usadel 2014)](https://paperpile.com/c/0nC3Wy/guhds) to remove residual adapters and select reads by quality and length. Read linear mapping to the human genome was performed with HISAT2 v2.0.4[(Kim, Langmead, and Salzberg 2015)](https://paperpile.com/c/0nC3Wy/bxK55).

CirComPara implements circular to linear proportion (CLP) as described in [(Cheng, Metge, and Dieterich 2016)](https://paperpile.com/c/0nC3Wy/ojclF):

$$CLP=\frac{circularreads}{circularreads+linearreads}$$

where *circular reads* are backspliced read count and *linear reads* are the count of linearly spliced reads at the backsplice junctions.

CirComPara’s non-default parameters used for analyses:

ADAPTER SEQUENCE = Trimmomatic file TruSeq3-PE-2.fa;

PREPROCESSOR = Trimmomatic,

TOGGLE_TRANSCRIPTOME_RECONSTRUCTION = 'False'; LINEAR_EXPRESSION_METHODS = 'stringtie';

CIRCRNA_METHODS = "testrealign, dcc, ciri, circexplorer2_star, findcirc, circexplorer2_segemehl, circexplorer2_bwa, circexplorer2_tophat, circrna_finder";

PREPROCESSOR_PARAMS = "MAXINFO:40:0.5 LEADING:20 TRAILING:20 SLIDINGWINDOW:4:30 MINLEN:50 AVGQUAL:30";

HISAT2_EXTRA_PARAMS = "--rna-strandness RF ";

STRINGTIE_PARAMS = '--rf';

BWA_PARAMS = ['-T', '19', '-c', '1'];

SEGEMEHL_PARAMS = ['-M','1', '-D', '0'];

TOPHAT_PARAMS = ['--zpacker', 'pigz', '--max-multihits', '1', '--library-type', 'fr-firststrand'];

STAR_PARAMS = ['--outFilterMultimapNmax', '1', '--outSJfilterOverhangMin', '15', '15', '15', '15', '--alignSJoverhangMin', '15', '--alignSJDBoverhangMin', '15', '--seedSearchStartLmax', '30', '--outFilterScoreMin', '1', '--outFilterMatchNmin', '1', '--outFilterMismatchNmax', '2', '--chimSegmentMin', '15', '--chimScoreMin', '15', '--chimScoreSeparation', '10', '--chimJunctionOverhangMin', '15'];

MIN_READS = 2;

MIN_METHODS = 2;

DCC_EXTRA_PARAMS = ['-fg', '-M', '-F', '-Nr', 1, 1];

TESTREALIGN_PARAMS = ['-q', 'median_10'];

FINDCIRC_EXTRA_PARAMS = ['--best-qual', '40'];

FIX_READ_HEADER = 'True';

SAM_SORT_MM = '6G';

CIRC_PE_MAPPING = 'True'

**CircRNA quantification by qRT-PCR**

After lymphoprep, mononuclear cells were frozen for RNA isolation in Trizol (ThermoFisher Scientific). After thawing, RNA was extracted from MNCs using the miRNeasy Mini or Micro Kit (Qiagen) in combination with on-column DNase I digestion (RNase-Free DNase set, Qiagen) according to manufacturer’s instructions. RNA concentrations were measured by Nanodrop (ThermoFisher Scientific) or Qubit RNA HS Assay (Invitrogen). cDNA synthesis was performed after an additional in-solution gDNase elimination step to eliminate residual genomic DNA (Heat&Run gDNA removal kit, ArcticZymes), using the 5x PrimeScript RT Master Mix (Takara Bio Europe S.A.S.) in a final volume of 12.5 µL. cDNA was diluted until a final concentration of 2.38 ng cDNA/µL.

Selection of targets for qPCR validation, was based on the significance and specificity (e.g. only present in JMML patients) of the differential expression, feasibility of primer development and previous literature concerning the role of the targets. Primers sequences were in-house developed using the NCBI primer pick tool and primer3 (https://bioinfo.ut.ee/primer3-0.4.0/). All primers for transcript evaluation were purchased at IDT Technologies. Primer efficiency was calculated by the LinRegPCR software (AMC, University of Amsterdam, the Netherlands), and approved if efficiency ranged between 85% and 115%.

qPCR reactions were carried out in 96-well plates using 0.3 µM primers, 2x Takyon Low ROX SYBR 2X MasterMix (Eurogentec), 2.38 ng cDNA and H2O (Sigma-Aldrich) in a 10 µL reaction. Samples were run in duplicate after a heat-activation step (3 min 95 °C) by a 2-step real-time protocol of 45 cycles (95 °C 15 sec, 60 °C 60 sec) on a Viia7 analyzer (ThermoFisher), combined with melting curve analysis (65 °C to 95 °C, gradually increasing with 0.5 °C/5 sec). Cq thresholds were automatically determined by the QuantStudio™ Real-Time PCR Software. Because of scarcity of JMML RNA (rare disease in very young children) and the multitude of targets we wanted to validate, we opted to perform qPCR experiments in duplicate with defined criteria. The maximum discrepancy between replicates we allowed varied between Cq values. These values were for Cq < 30: 0.5 Cq, for Cq > 30 and < 33: 1 Cq, for Cq > 33 and < 36 = 1.5 Cq and for Cq > 36 = 2 Cq.

Housekeeping genes for normalization were a priori selected in a pilot study as follows: The expression of eight housekeeping genes (GAPD, HMBS, HPRT1, RPL13A, SDHA, TBP, UBC and YWHAZ) and Alu repeats was investigated in 11 cell lines, chosen based on a broad genetic repertoire and pediatric origin. Adhering to a strict M-value ≤ 0.5 and V-value < 0.15, six out of eight housekeeping genes (ranged from highest to lowest stability: TBP, GAPD, HPRT1, HMBS, SDHA and YWHAZ) were advised for gene of interest normalization. The three most stable housekeeping genes GAPD, HPRT1 and TBP (V-value 0.202, respective M-values 0.45, 0.45 and 0.57) were selected.

Data analysis was performed according to state-of-the-art methods (Hellemans, J. et al. Genome Biol 2007). Briefly, Cq values generated for each target were corrected for primer pair efficiency and expressed as relative quantities (RQ). Normalized relative quantities (NRQ) were calculated by normalizing RQ values against the expression of housekeeping genes GAPD, HPRT1 and TBP (NRQ). To allow inter-run comparison, calibrated NRQ values (CNRQ) were generated by considering the expression of a single inter-run calibrator (IRC), evaluated in each run by the respective primer pair.

| Supplementary FiguresSupplementary Figure 1. Genes with at least 10 circular isoforms. A) Number of isoforms for each gene; B) Heatmaps of circRNA expression for each gene (expression in logarithmic scale).  **A B**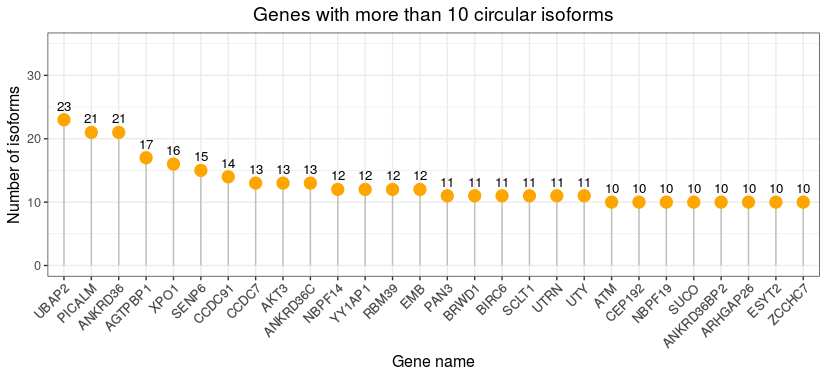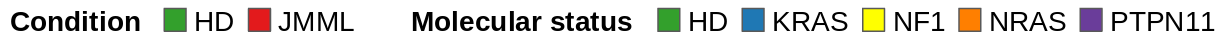  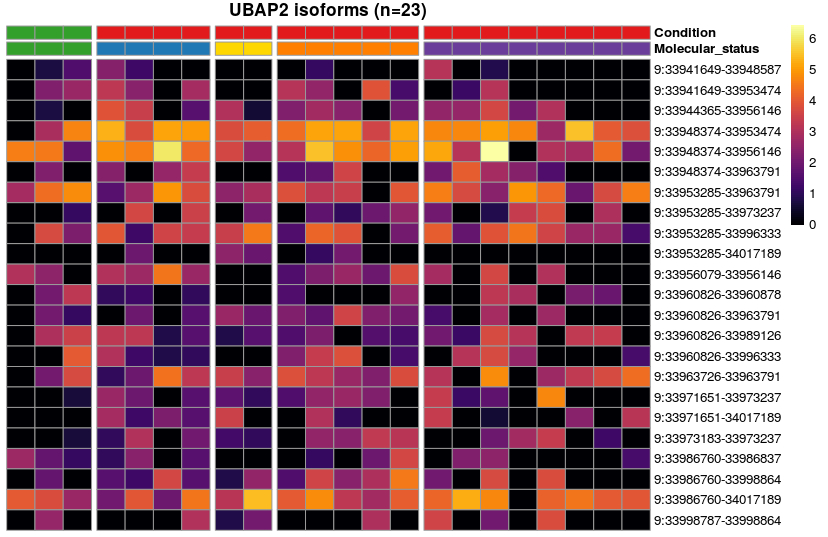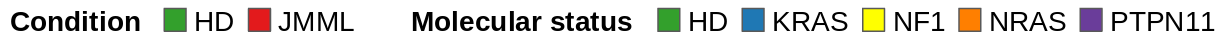 | | |
| --- | --- | --- |
| 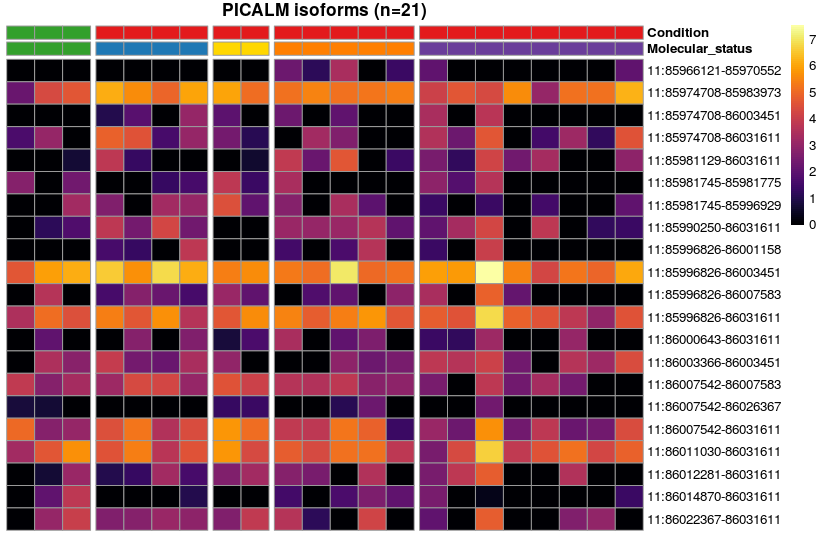 | 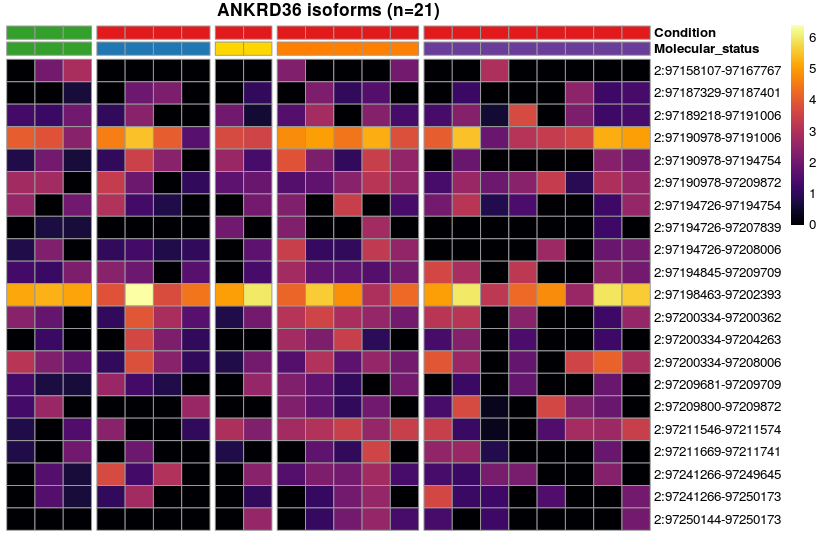 | 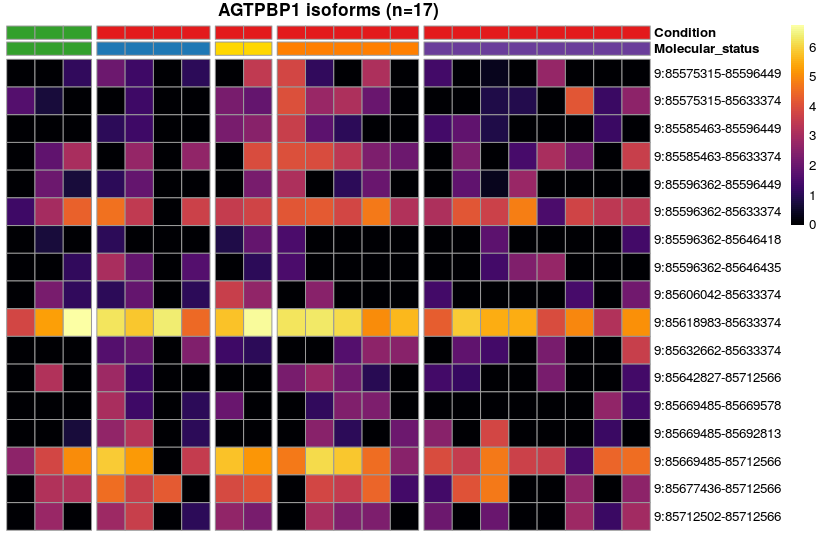 |
| 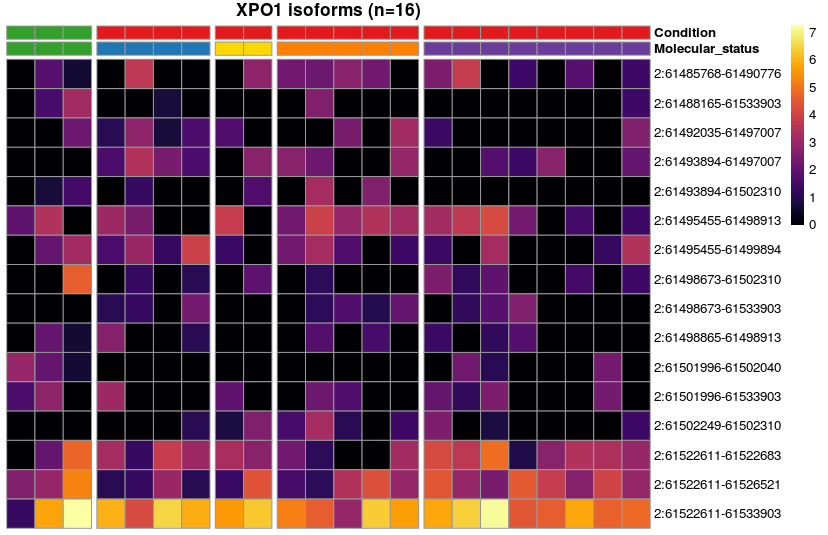 | 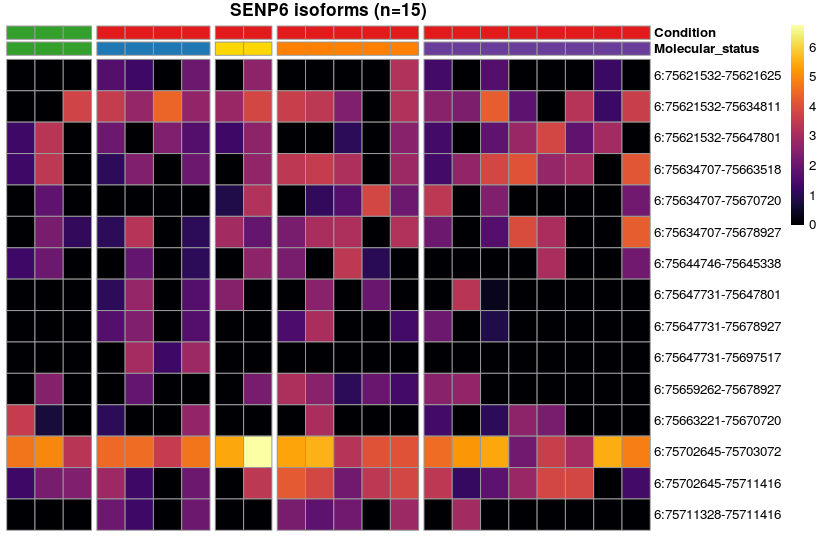 | 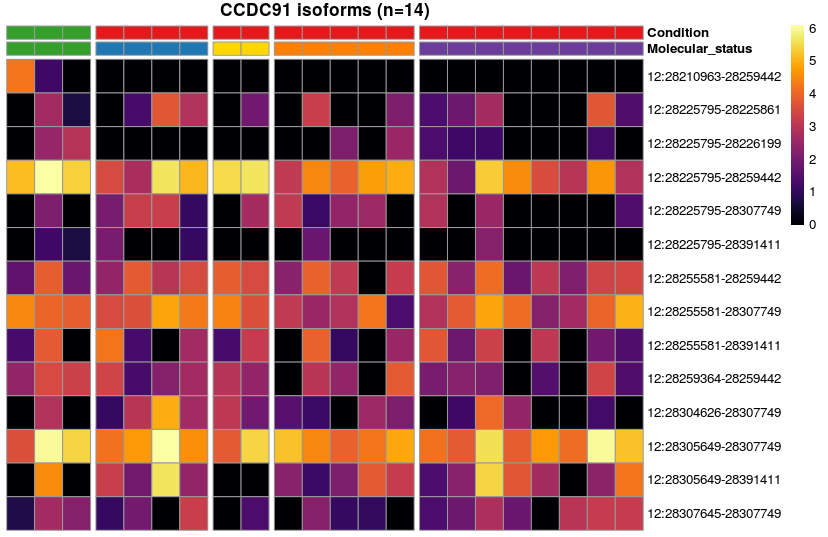 |
| 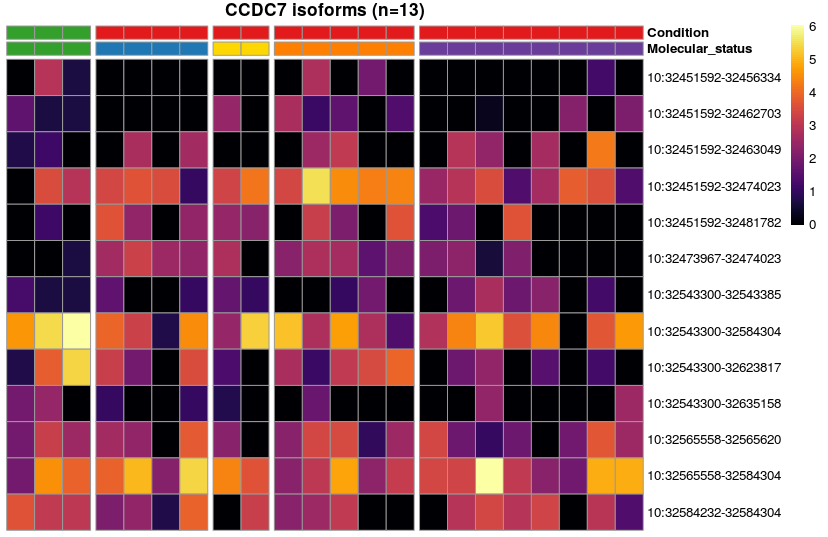 | 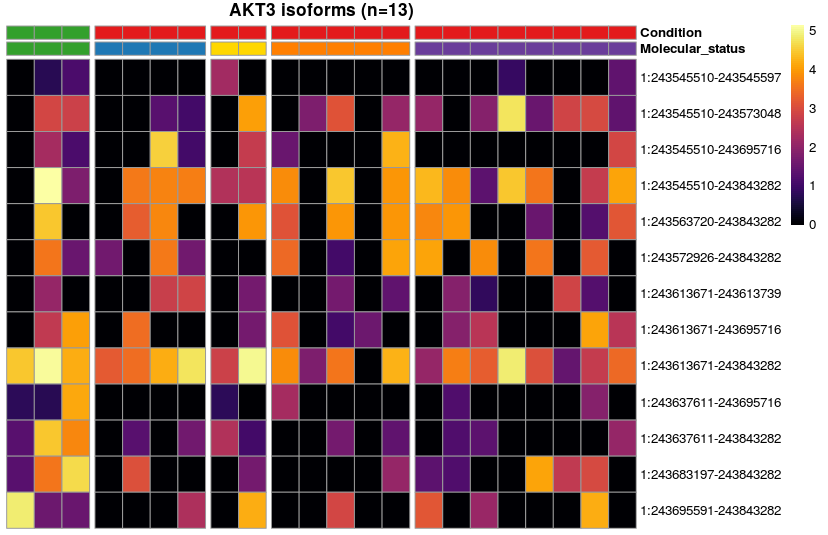 | 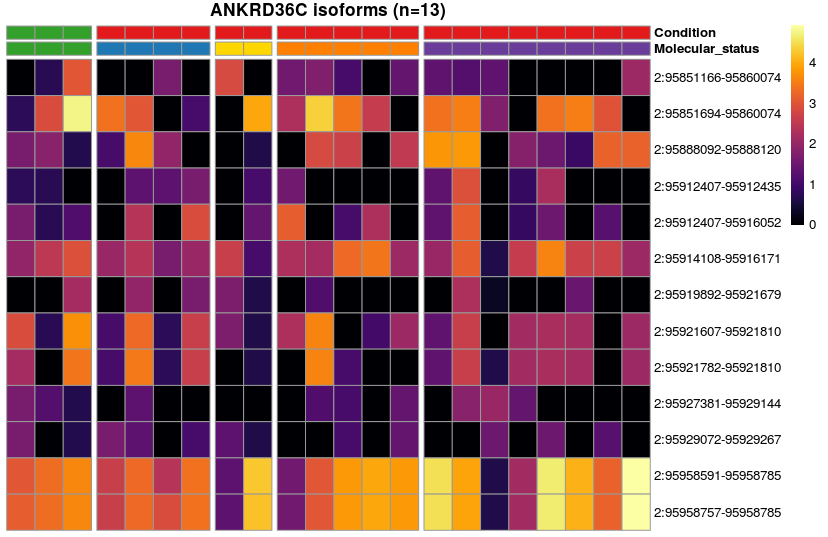 |
| 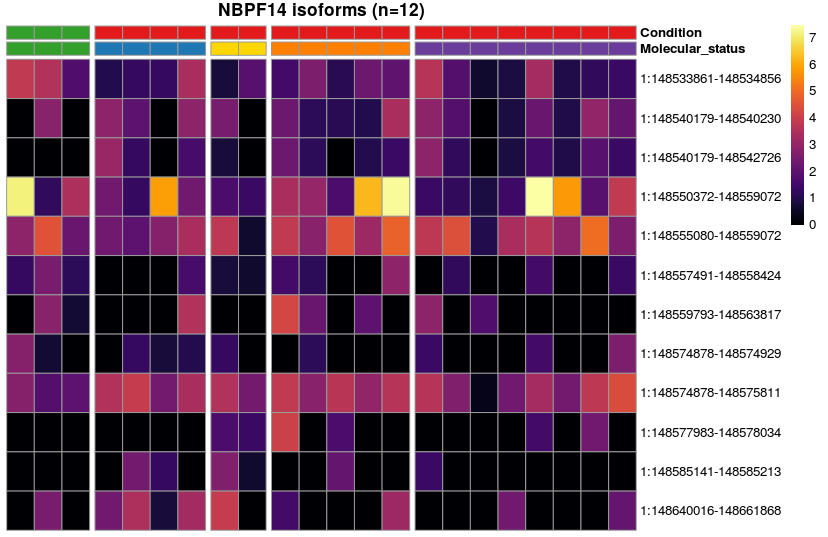 | 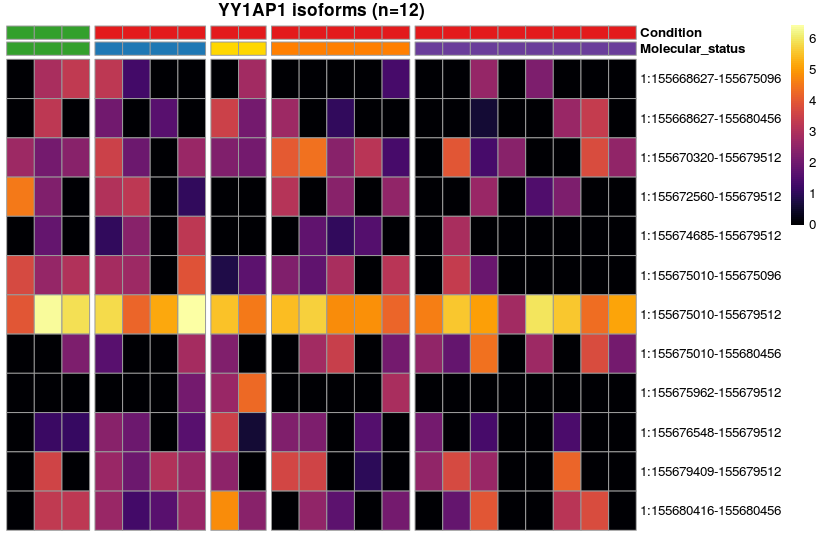 | 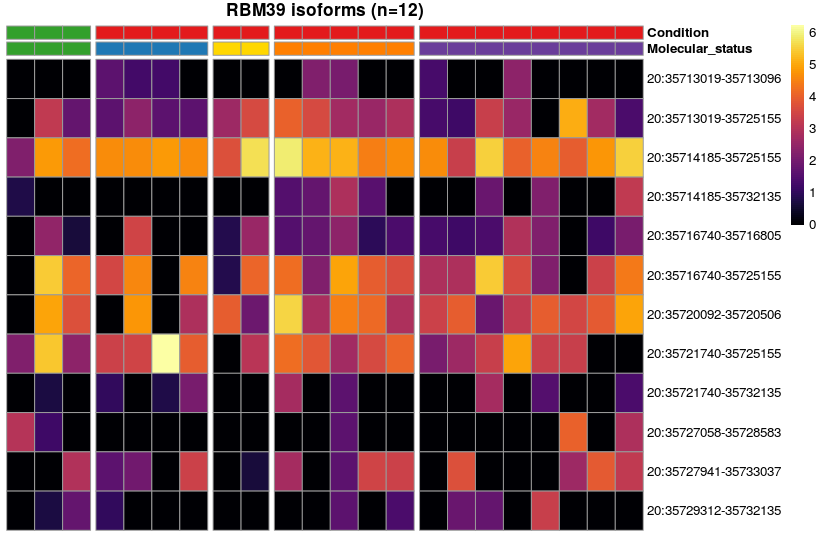 |
| 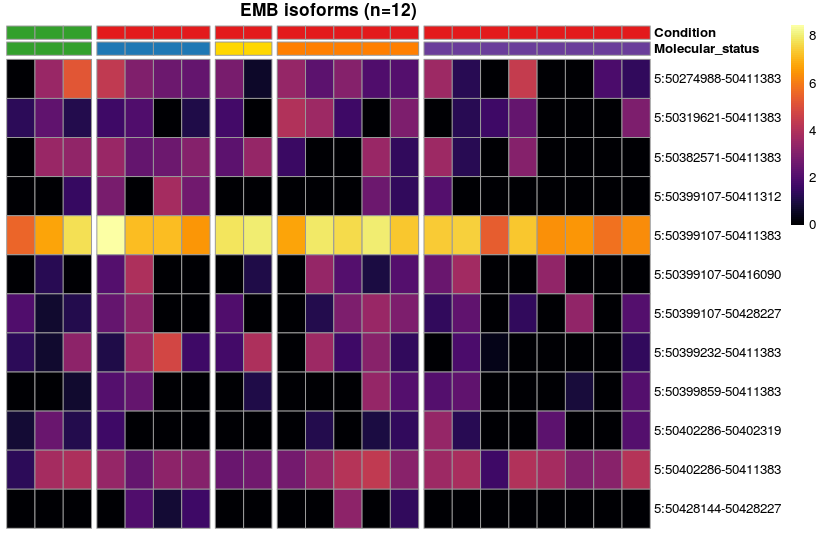 | 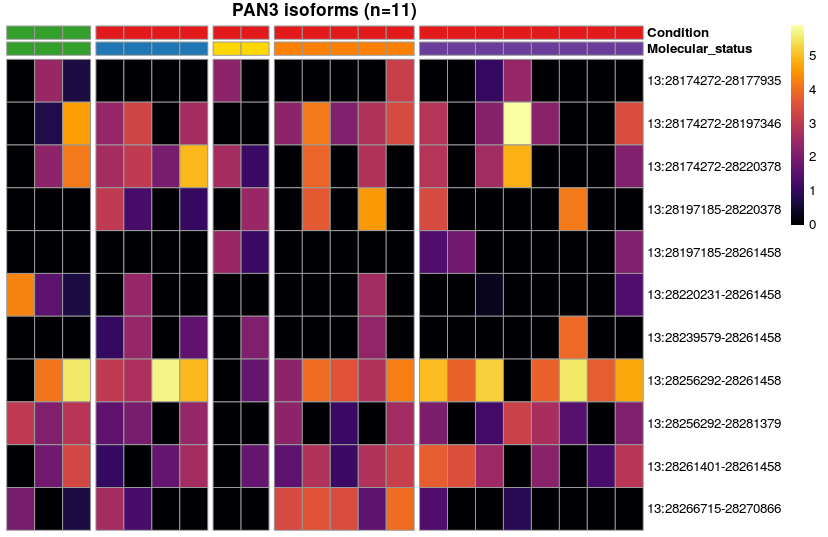 | 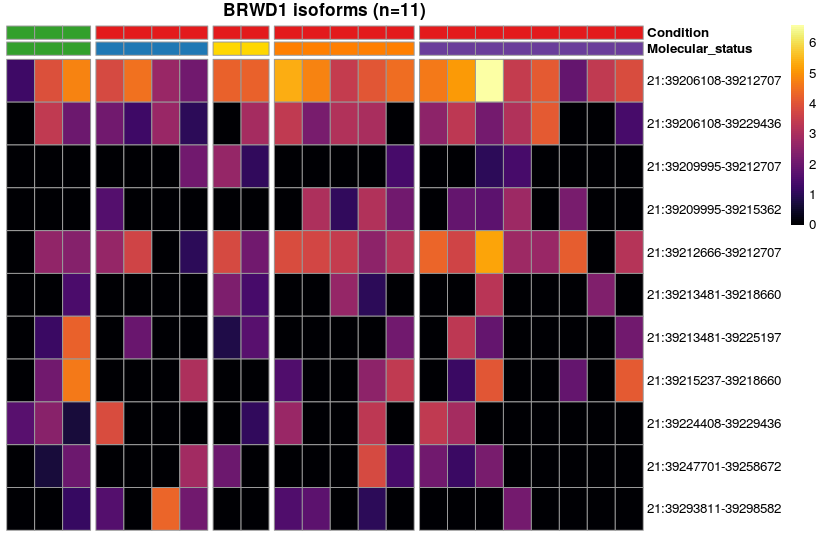 |
| 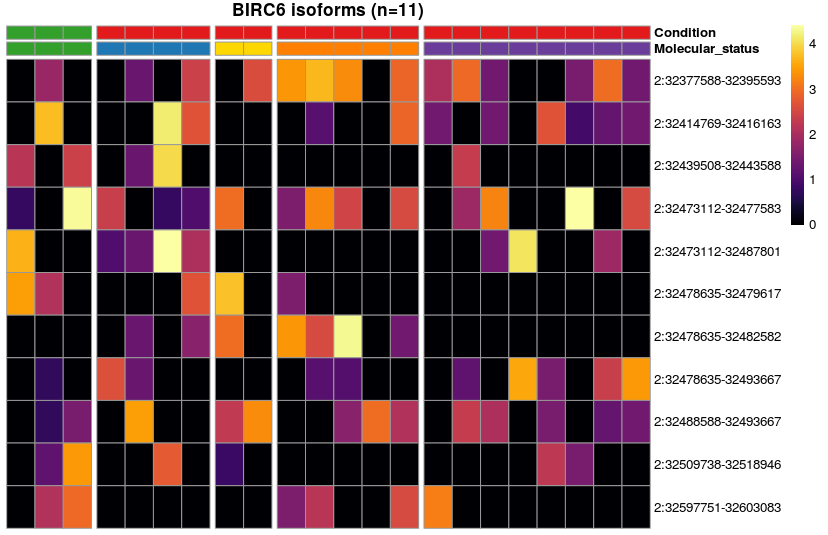 | 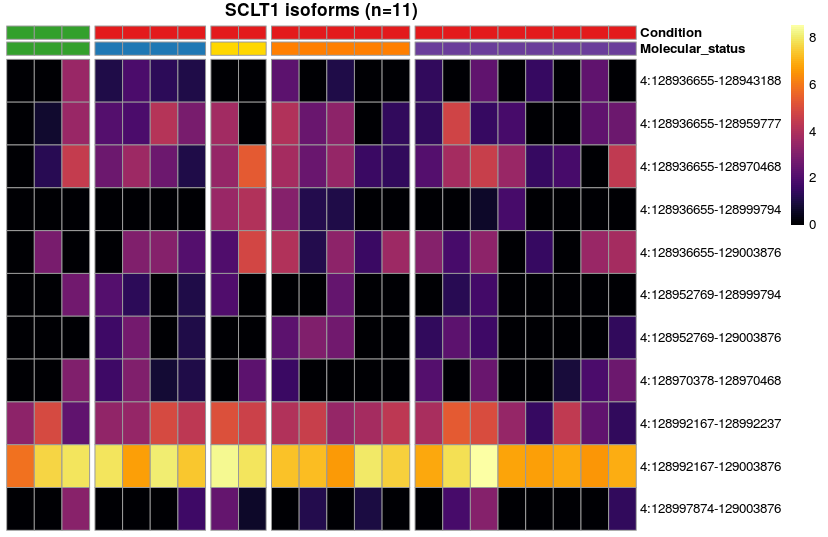 | 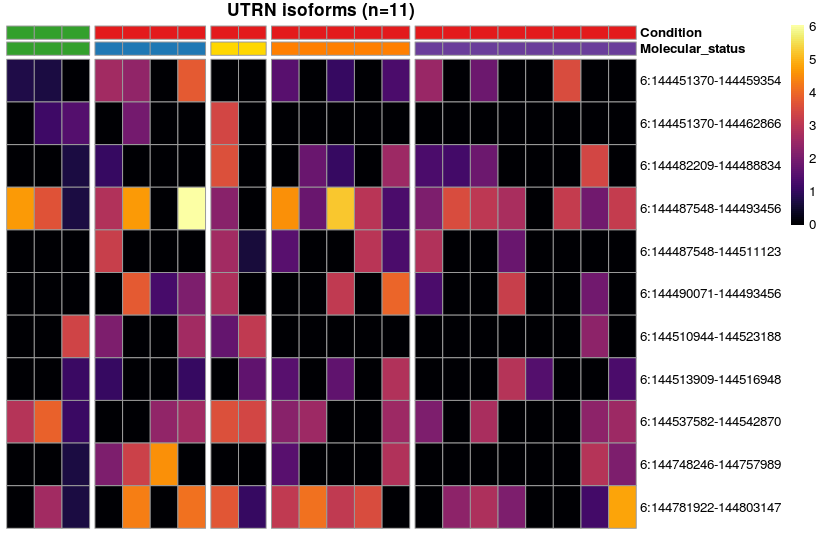 |
| 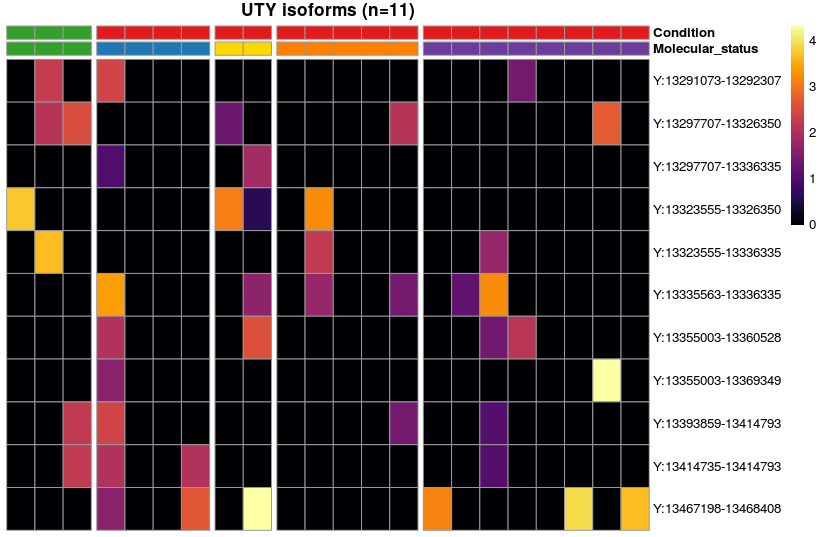 | 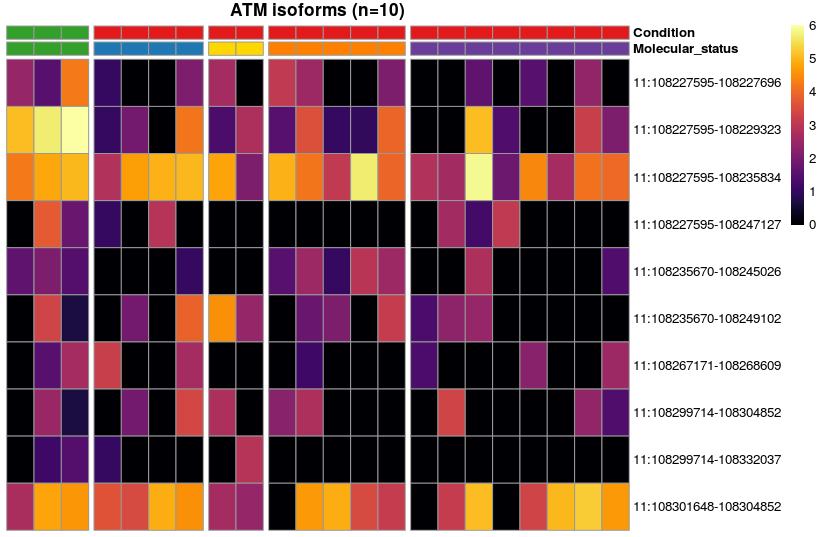 | 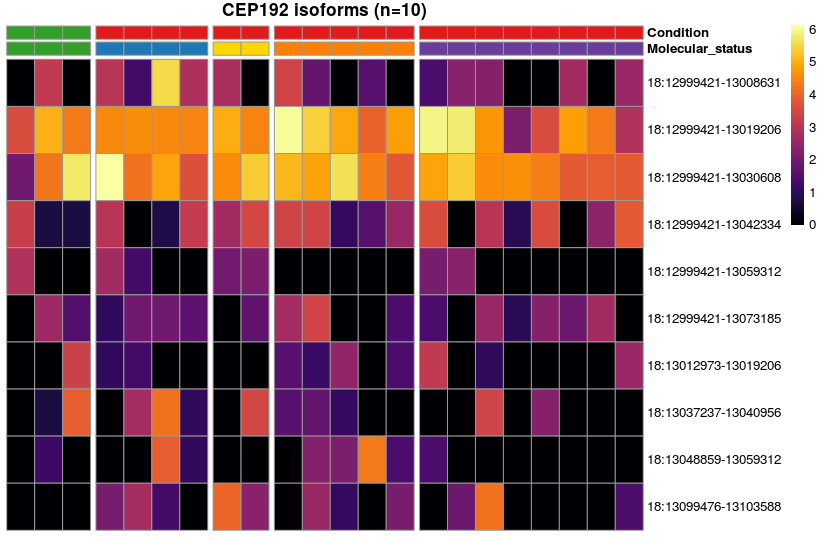 |
| 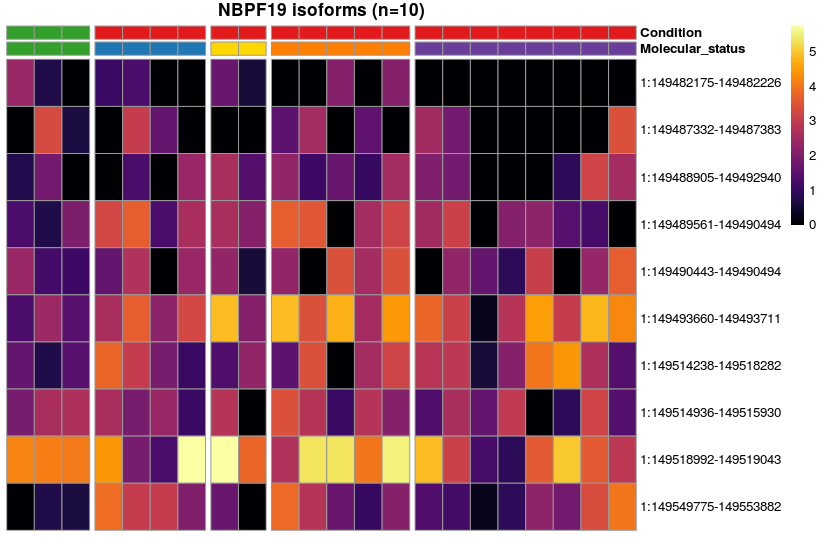 | 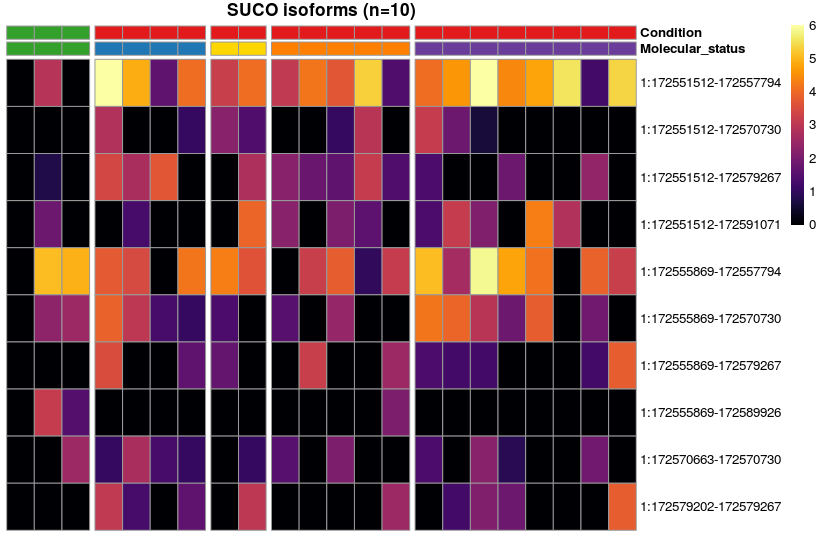 | 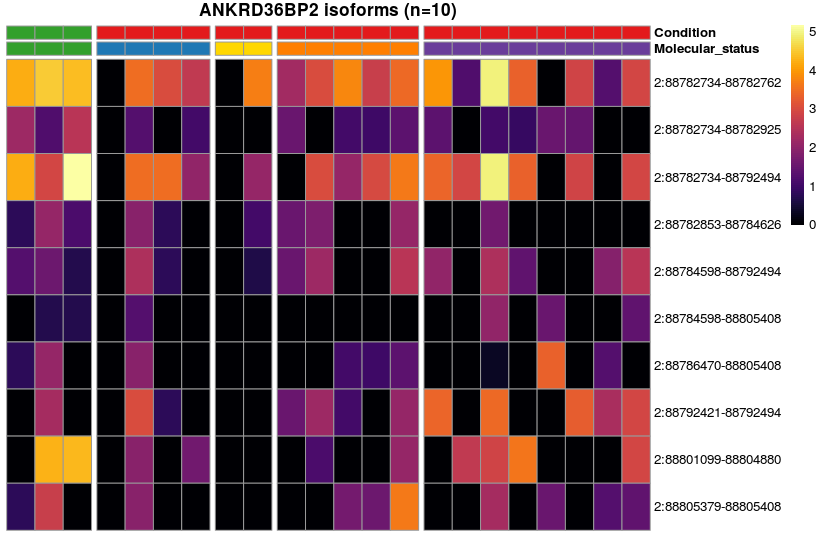 |
| 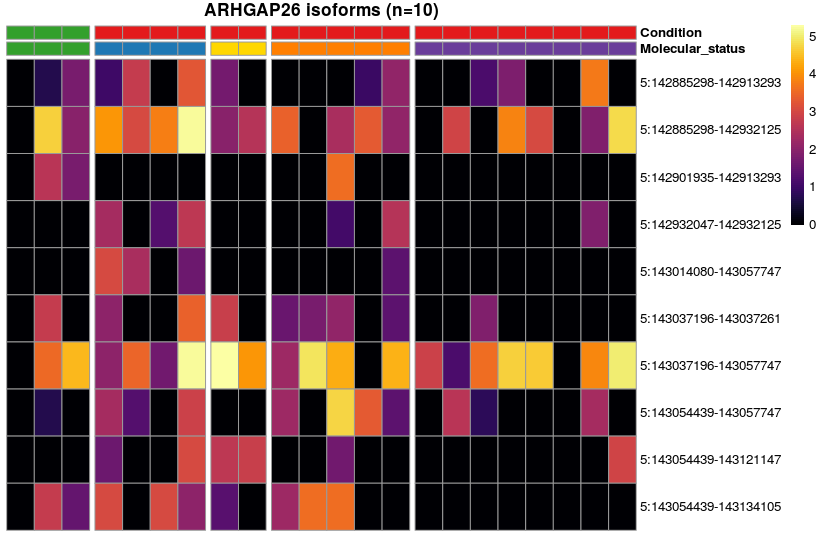 | 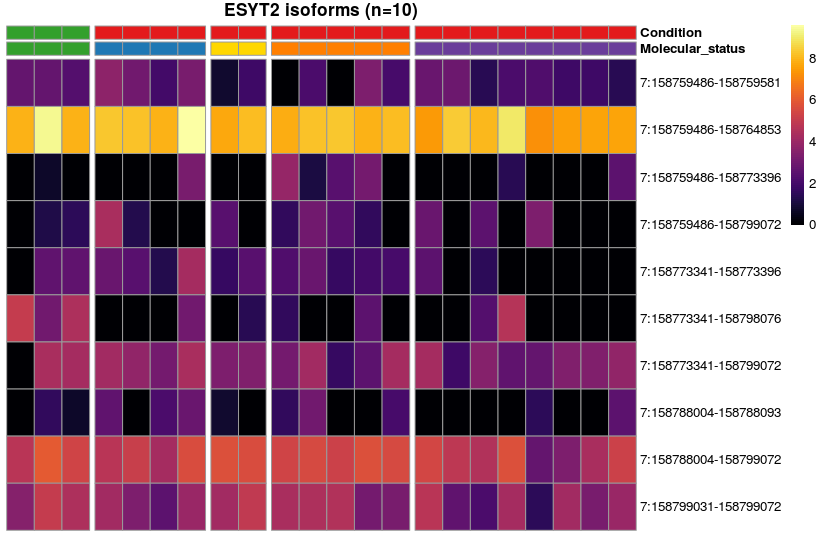 | 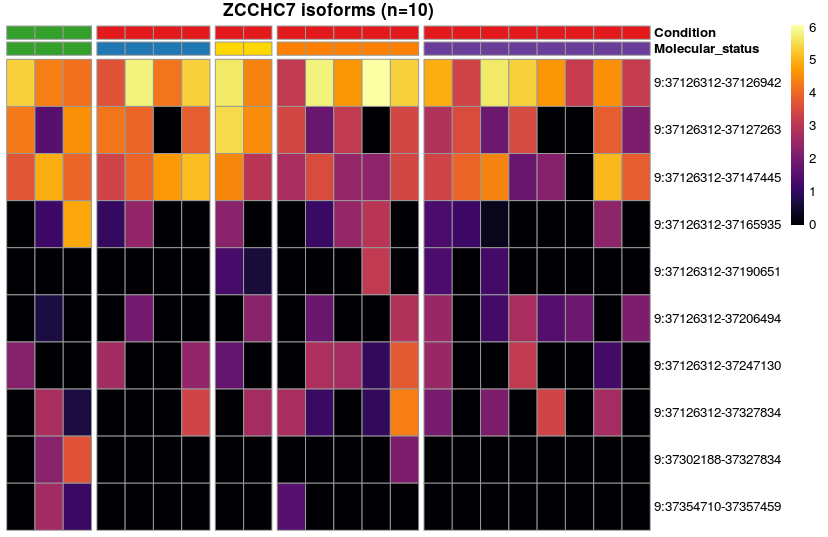 |

## Supplementary Figure 2. Expression variation in 21 JMML cases of the circRNA panel quantified by qRT-PCR.

QRT-PCR quantifications of 20 circRNAs, with differential expression between JMML and HD according to RNA-seq data, in 21 JMML (red dots) and 6 age-matched HD samples (green dots). CircRNA quantification in not less than 17 patients was obtained for all the circRNAs; p-values (two-tailed) were calculated by the Mann-Whitney U test.


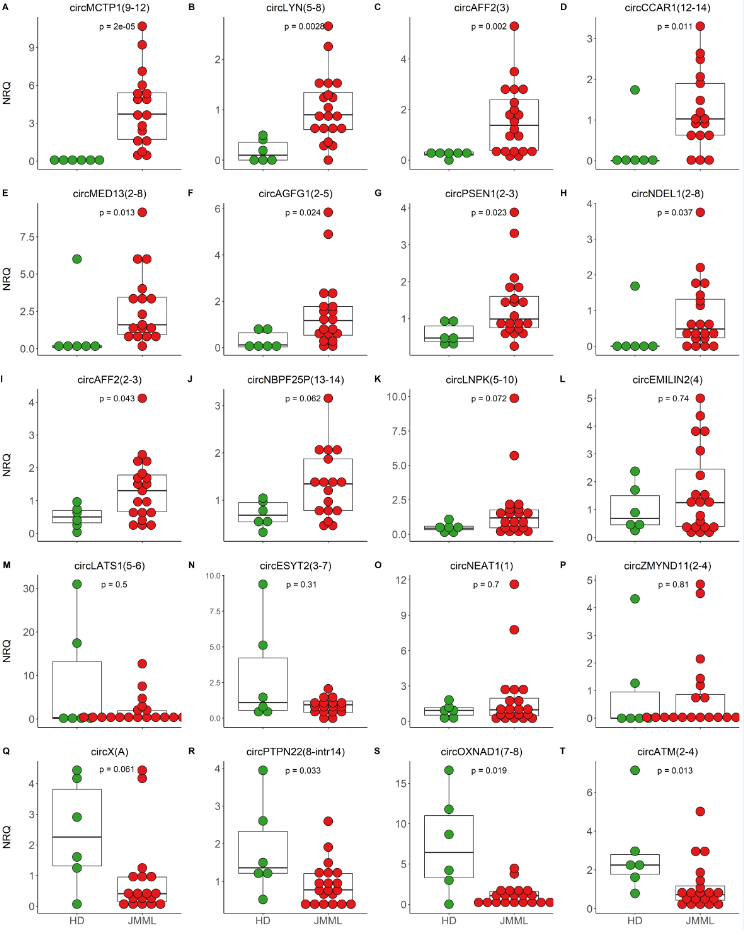


## Supplementary Figure 3. Predicted circRNA-miRNA-gene network involving circRNAs most deregulated in JMML.

CircRNAs up-regulated and down-regulated in JMML are shown in red and green circles, respectively; circRNAs are linked to miRNAs (diamonds) according to binding sites predicted by both miRanda and PITA tools; in addition to miRNAs expressed in JMML[^13^](https://paperpile.com/c/0nC3Wy/HaL7) (in bold), recently identified miRNAs with at least three predicted binding sites are also reported; size of circRNA-miRNA edges is proportional to the number of the predicted binding sites for that miRNA in that circRNA (range 1-8); validated miRNA target genes resulting from Mienturnet enrichment (strong MiRTarBase categories, FDR<0.1) are shown as squares, with fill color indicating the LFC of the gene expression variation comparing JMML with HD.


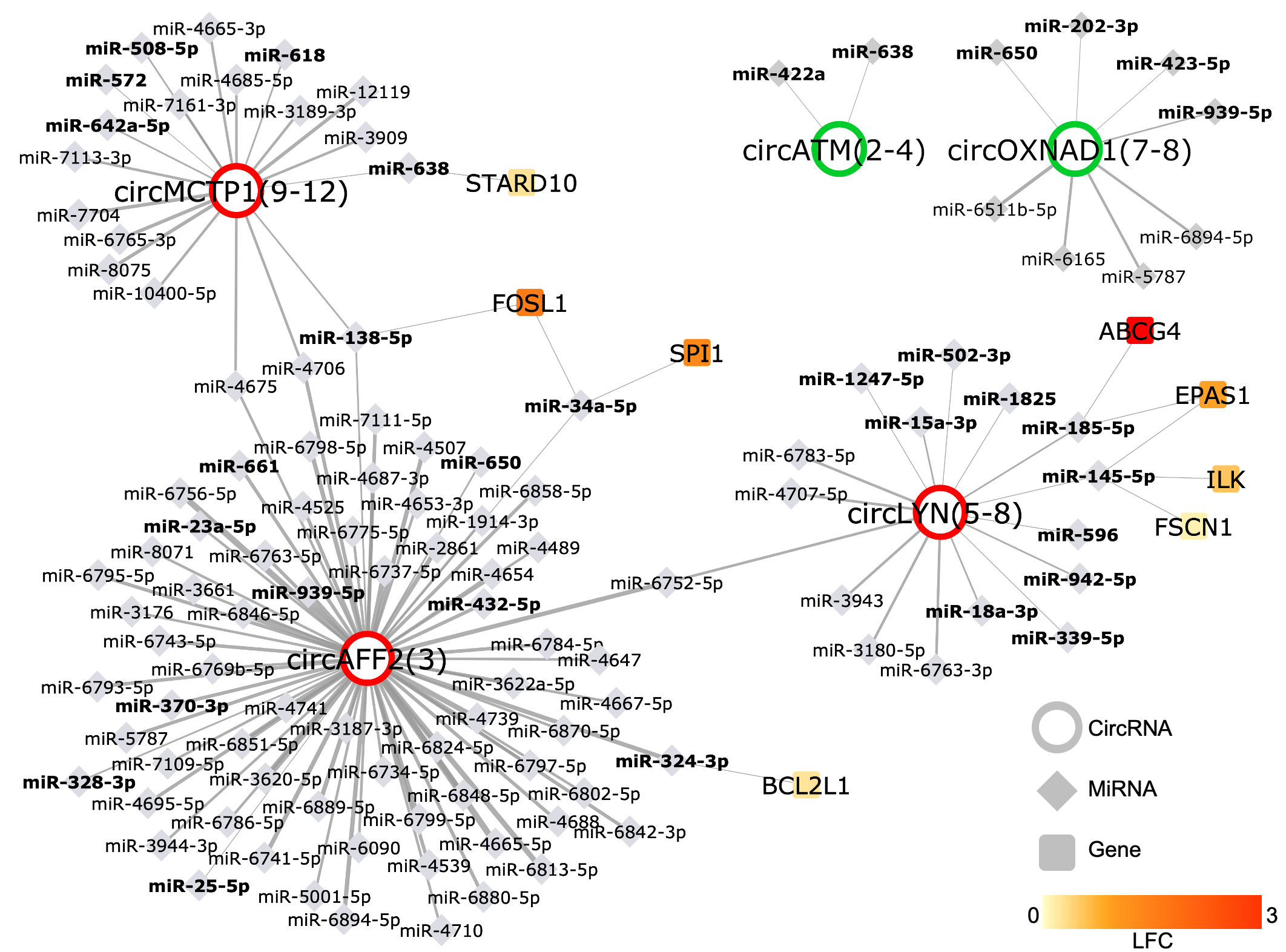


## Supplementary Figure 4. Expression variation in 21 JMML of different subtypes of the circRNA panel quantified by qRT-PCR.

QRT-PCR expression data of 14 circRNAs in 21 JMML of 5 molecular subtypes (KRAS, NRAS, PTPN11 and NF1 mutated, and quintuple negative (5N)), and 6 age-matched HD samples. CircRNA quantification in not less than 17 patients was obtained for all the circRNAs; differential expression of KRAS, NRAS and PTPN11 groups compared with HD (Kruskal-Wallis test) and to each other was tested with Mann-Whitney U test; only p-values < 0.1 are shown for the corresponding pairwise comparison.

##
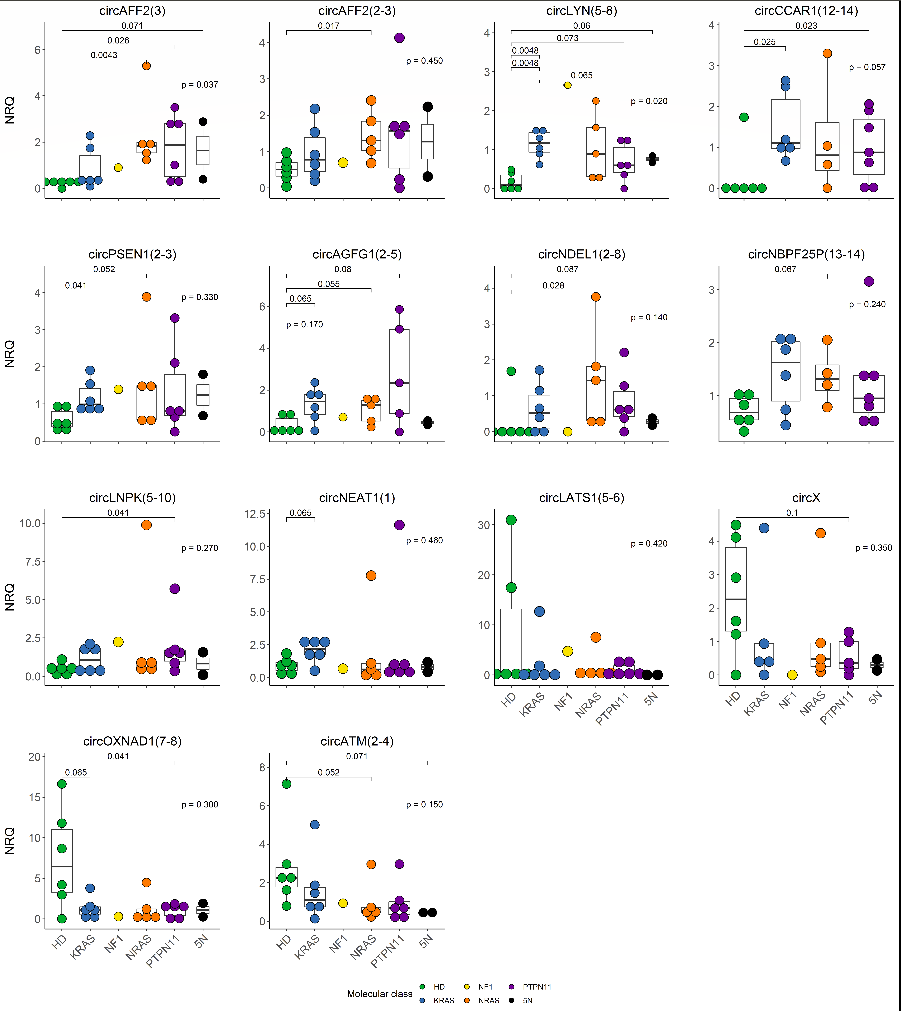

Supplement: Supplementary file 1 [file Table_1.DOCX]
